# Serendipitous Identification of Azine Anticancer Agents Using a Privileged Scaffold Morphing Strategy

**DOI:** 10.3390/molecules29071452

**Published:** 2024-03-24

**Authors:** Silvia Cesarini, Ilaria Vicenti, Federica Poggialini, Silvia Filippi, Eleonora Mancin, Lia Fiaschi, Elisa De Marchi, Federica Giammarino, Chiara Vagaggini, Bruno Mattia Bizzarri, Raffaele Saladino, Elena Dreassi, Maurizio Zazzi, Lorenzo Botta

**Affiliations:** 1 Department of Biological and Ecological Sciences, University of Viterbo, Via S.C. De Lellis s.n.c., 01100 Viterbo, Italy; c.cesarinisilvia@gmail.com (S.C.); silvia.filippi@unitus.it (S.F.); eleonora.mancin98@gmail.com (E.M.); elisa.demarchi@unitus.it (E.D.M.); bm.bizzarri@unitus.it (B.M.B.); saladino@unitus.it (R.S.); 2 Department of Medical Biotechnologies, University of Siena, 53100 Siena, Italy; ilariavicenti@gmail.com (I.V.); lia.fiaschi@unisi.it (L.F.); federica.giammari@gmail.com (F.G.); maurizio.zazzi@gmail.com (M.Z.); 3 Department of Biotechnology, Chemistry, and Pharmacy (DBCF), University of Siena, 53100 Siena, Italy; poggialini5@student.unisi.it (F.P.); chiara.vagaggini@student.unisi.it (C.V.); elena.dreassi@unisi.it (E.D.)

**Keywords:** privileged scaffold, triazines, pyrimidines, pyridines, scaffold morphing, anticancer activity

## Abstract

The use of privileged scaffolds as a starting point for the construction of libraries of bioactive compounds is a widely used strategy in drug discovery and development. Scaffold decoration, morphing and hopping are additional techniques that enable the modification of the chosen privileged framework and better explore the chemical space around it. In this study, two series of highly functionalized pyrimidine and pyridine derivatives were synthesized using a scaffold morphing approach consisting of triazine compounds obtained previously as antiviral agents. Newly synthesized azines were evaluated against lymphoma, hepatocarcinoma, and colon epithelial carcinoma cells, showing in five cases acceptable to good anticancer activity associated with low cytotoxicity on healthy fibroblasts. Finally, ADME in vitro studies were conducted on the best derivatives of the two series showing good passive permeability and resistance to metabolic degradation.

## 1. Introduction

The concept of the privileged scaffold was introduced by Evans in 1988 [1] and represents a molecular framework able to bind more than one receptor and act as agonist or antagonist, when conveniently functionalized. Among all the drug discovery strategies to harness privileged scaffolds, decoration with opportune substituents is a good technique that allows the exploration of the biologically relevant chemical space around the chosen promising framework [2]. In addition, scaffold morphing [3] and hopping [4] are useful procedures to replace the central core structure of an active molecule with the aim of identifying new bioactive entities with increased pharmacodynamic and pharmacokinetic properties.

**Figure 1:**
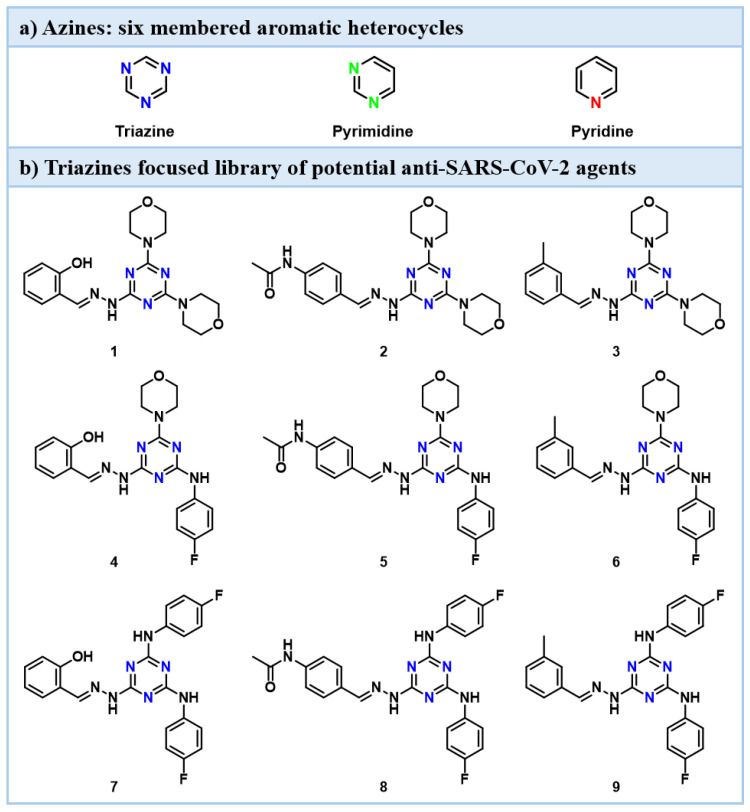
Chemical structure of three azine basic nuclei (**a**) and a focused library of triazine derivatives (**1**–**9**) synthesized in a previous work (**b**) [9].

Basic nuclei of great interest are azines, six membered aromatic heterocycles that contain at least one nitrogen atom. Therefore, triazines, pyrimidines and pyridines (Figure 1a), can be considered privileged scaffolds [5,6] since their high prevalence in commercially available drugs [7] and biologically relevant biomolecules (e.g., enzymes cofactors, nucleobases) [8].

In the last few years, our research group has been involved in the synthesis and decoration of privileged scaffolds [9]. Concerning triazines, our team recently published a paper [9] focused on the synthesis and evaluation of compounds **1**–**9** against severe acute respiratory syndrome coronavirus type 2 (SARS-CoV-2) (Figure 1b). Unfortunately, from this library of molecules only compound **1** emerged as able to inhibit SARS-CoV-2 replication with an IC_50_ (half-maximal compound concentration inhibiting the 50% of virus replication) value of 12.1 micromolar (mM).

Additional studies aimed at addressing a potential antiviral activity of derivatives **1**–**9** were conducted on several pathogens, highlighting a low or absent effect on human immunodeficiency (HIV-1), Dengue (DENV) and West Nile viruses (WNV). The exception registered was compound **4** that showed potency on flaviviruses DENV and WNV in the low micromolar range (2.0 and 1.1 µM, respectively).

Surprisingly, viability assays conducted on uninfected lymphoma (H9), hepatocarcinoma (Huh7), and colon epithelial carcinoma (Caco-2) cells showed a cytotoxic effect of the synthesized molecules. This behavior prompts us to test compounds **1**–**9** on human normal fibroblasts FB789 and calculate the tumor selectivity indexes (TSIs) as the ratio between half-maximal compound cytotoxic concentration (CC_50_) on FB789 and in turn CC_50_ in H9, Huh7 and Caco-2 (Table 1). 

From all the evaluations made, derivatives **4** and **7** seemed to be interesting hit compounds, since they showed low micromolar antitumor activity on Huh7 (1.2 and 3.5 TSIs for derivatives **4** and **7**, respectively) and H9 (5.5 and 3.5 TSIs for triazines **4** and **7**, respectively) and a submicromolar effect on Caco-2 for compound **7** (8.8 TSI) (Table 1, entries 4 and 7). Moreover, from the cytotoxicity on FB789 cells point of view, triazine **3** highlighted the best profile compared to other compounds of the series (Table 1, entry 3). For these reasons, we decided to begin a study based on these results serendipitously obtained with triazine molecules aimed at the identification of potential antineoplastic agents for lymphoma, hepatocarcinoma and colorectal cancer. Hence, we decided to apply a scaffold morphing approach to replace the triazine core with pyrimidine and pyridine ones and to decorate these azine nuclei with the substituents that showed the best pharmacological performance in the previous study [9]. In detail, morpholine and 4-fluoroaniline were used as the pharmacophoric functionalities directly linked to the central six membered heterocycle. Salicyl and 3-methylphenyl hydrazones were chosen to complete the panel of substituents for the novel highly functionalized pyrimidine and pyridine products, since they are characteristic of the above-mentioned derivatives **3**, **4** and **7**. In the present study, two small libraries of a total twelve compounds were synthesized and were evaluated against H9, Huh7, and Caco-2 lines. Finally, the in vitro absorption, distribution, metabolism, and excretion (ADME) properties of selected derivatives were screened to define the most promising scaffold and the best highly functionalized derivative synthesized.

## 2. Results and Discussion

**Scheme 1:**
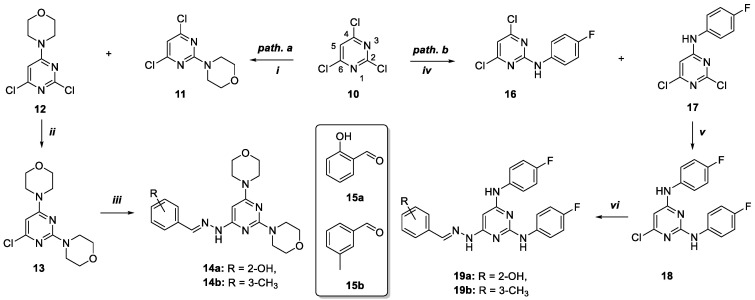
Synthesis of pyrimidine derivatives **14a**–**b** (pathway a) and **19a**–**b** (pathway b). Reagents and conditions: (i) dichloromethane (CH_2_Cl_2_), morpholine, *N*,*N*-Diisopropylethylamine (DIPEA), 0 °C, 5 h; (ii) 1,4-dioxane, morpholine, HCl, MW, 110 °C, 40 min; (iii) 1. 1,4-dioxane, NH_2_NH_2_, MW, 100 °C, 4 h, 2. methanol (MeOH), **15a**,**b**, acetic acid, 25 °C, 24 h; (iv) CH_2_Cl_2_, 4-fluoroaniline, DIPEA, 0 °C, 5 h; (v) 1,4-dioxane, 4-fluoroaniline, HCl, MW, 110 °C, 40 min; (vi) 1. 1,4-dioxane, NH_2_NH_2_, MW, 100 °C, 4 h, 2. MeOH, **15a**,**b**, acetic acid, 25 °C, 48 h.

As depicted in Scheme 1 pathway a, highly functionalized trisubstituted pyrimidine derivatives **14a**,**b** were obtained in a three steps sequence starting from 2,4,6-trichloro pyrimidine **10** [10]. The latter was transformed into compounds **11** and **12** in 19 and 68% yield through reaction with morpholine in the presence of *N*,*N*-diisopropylethylamine (DIPEA) at 0 °C. The higher yield of the C6-substituted **12**, compared to the C-2 functionalized **11**, is due to steric hinderance exerted by the lone pairs of the N1 and N3 atoms that discourage the access of the nucleophile in the C2 position of the pyrimidine ring [11].

To distinguish between the two regioisomers, and for the final structure assignment, 2D NMR Nuclear Overhauser Effect Spectroscopy (NOESY) experiments were conducted [Supplementary Materials S1]. In derivative **12** it was possible to identify the interaction between the proton in position 5 of the pyrimidine ring and protons on the carbon atom adjacent to morpholine nitrogen, which was absent in **11**.

Then, **12** was subjected to a second substitution reaction always in the presence of morpholine at 110 °C under microwave (MW) irradiation to obtain Compound **13**. Finally, disubstituted pyrimidine **13** was reacted with hydrazine (NH_2_NH_2_) and subsequently with the opportune aldehyde, salicyl- and 3-methylphenyl-aldehydes **15a**,**b**, to give desired products **14a** and **14b** with 55 and 65% yield, respectively.

Highly functionalized pyrimidines **19a**,**b** were synthesized with a pathway similar to the above-described one (Scheme 1, path. b), using 4-flouroaniline for the first two aromatic nucleophilic substitutions instead of morpholine.

**Scheme 2:**
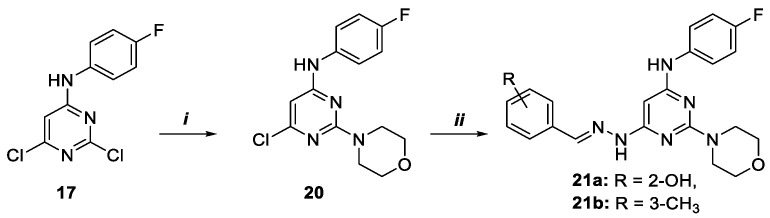
Synthesis of pyrimidine derivatives **21a**,**b**. Reagents and conditions: (i) ethanol (EtOH), morpholine, Et_3_N, 80 °C, 18 h; (ii) 1. 1,4-dioxane, NH_2_NH_2_, MW, 100 °C, 6 h, 2. MeOH, **15a**,**b**, acetic acid, 25 °C 48 h.

Pyrimidines **21a**,**b** were afforded starting from the monosubstituted intermediate **17** previously obtained (Scheme 2). The latter was easily turned into the final products through nucleophilic substitution with morpholine and the hydrazone two steps sequence installation.

**Scheme 3:**
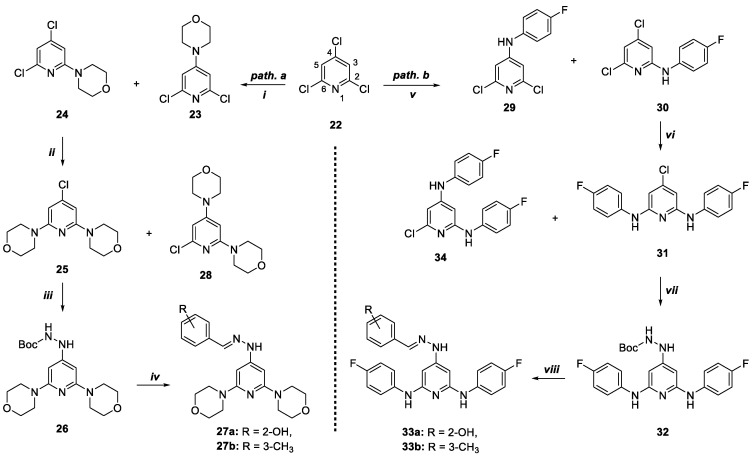
Synthesis of pyridine derivatives **27a**,**b** (pathway a) and **33a**,**b** (pathway b). Reagents and conditions: (i) THF, morpholine, Et_3_N, 65 °C, 18 h; (ii) dimethyl formamide (DMF), morpholine, DIPEA, 120 °C, 48 h; (iii) toluene, BocNHNH_2_, tris(dibenzylideneacetone)dipalladium (0) [Pd_2_(dba)_3_], 1,1′-Bis(diphenylphosphino)ferrocene (DPPF), cesium carbonate (Cs_2_CO_3_), MW, 150 °C, 10 min; (iv) 1. CH_2_Cl_2_, TFA, 25 °C, 1 h, 2. MeOH, **15a**,**b**, acetic acid, 25 °C, 24 h; (v) toluene, tetrakis(triphenylphosphine)-palladium (0) [Pd(PPh_3_)_4_], 4-fluoroanilina, Cs_2_CO_3_, 150 °C, 24 h; (vi) toluene, palladium(II) acetate [Pd(OAc)_2_], 2,2′-Bis(diphenylphosphino)-1,1′-binaphyl (BINAP), 4-fluoroaniline, Cs_2_CO_3_, 160 °C, 4 h; (vii) toluene, BocNHNH_2_, Pd_2_(dba)_3_, DPPF, Cs_2_CO_3_ MW, 150 °C, 10 min; (viii) 1. CH_2_Cl_2_, TFA, 25 °C, 1 h, 2. MeOH, **15a**,**b**, acetic acid, 25 °C, 24 h.

Pyridine derivatives were synthesized as follows. Starting from 2,4,6-trichloropyridine **22**, through nucleophilic substitution with morpholine in tetrahydrofuran (THF) and triethyl amine (Et_3_N) at 65 °C, a mixture of the two regioisomers **23** and **24**, bearing the amine substituent on C-4 and C-2, respectively, was obtained (Scheme 3, path. a). Structural assignment was made based on ^1^H NMR experiments, being **23** symmetric and showing only one peak for C3–C5 protons in the recorded spectrum (Supplementary Materials S2). From compound **24**, two regioisomeric pyridines, compounds **25** and **28**, were synthesized via morpholine nucleophilic substitution at 120 °C. Then, many attempts to directly introduce NH_2_NH_2_ on the heterocyclic ring were made, such as the use of high temperature, MW irradiation, and high concentration of NH_2_NH_2_, but the desired pyridine carrying the hydrazine functionality on C-4 was never isolated. Finally, with tert-butoxy carbonyl (Boc) protected hydrazine, namely tert-butyl carbazate (BocNHNH_2_), it was possible to decorate the C-4 position of the azine core [12]. When compound **26** was obtained, it was turned into the final trisubstituted products **27a**,**b** with 39 and 18% yield via acidic Boc removal in the presence of trifluoroacetic acid (TFA), and a reaction with the opportune aldehydes **15a**,**b** in a mixture of MeOH and a few drops of acetic acid at 25 °C.

With a similar route, the desired highly decorated pyridines **33a**,**b** were obtained (Scheme 3, path. B).

**Scheme 4:**
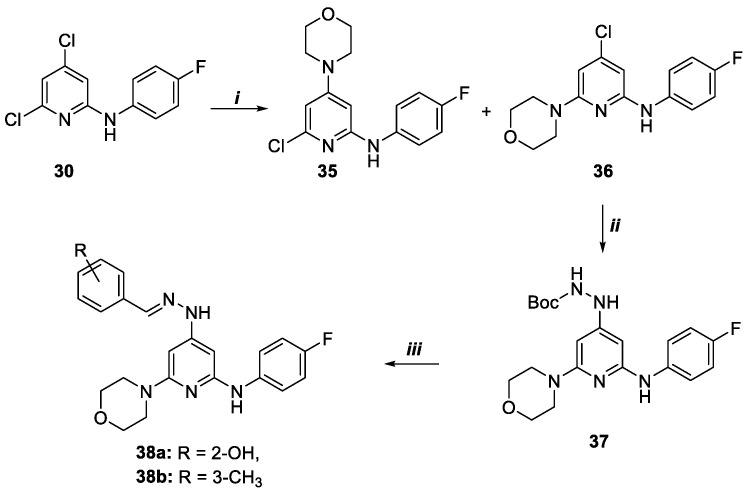
Synthesis of pyridine derivatives **38a,b**. Reagents and conditions: (i) 1,4-dioxane, morpholine, DIPEA, 160 °C, 10 h; (ii) toluene, BocNHNH_2_, Pd_2_(dba)_3_, DPPF, Cs_2_CO_3_ MW, 150 °C, 10 min; (iii) 1. CH_2_Cl_2_, TFA, 25 °C, 1 h, 2. MeOH, **15a**,**b**, acetic acid, 25 °C, 24 h.

Finally, compounds **38a**,**b** were obtained in a 37% and 33% yield with a three step sequence starting from monosubstituted pyridine **30** (Scheme 4). The latter was converted into the desired products through reaction with morpholine at 160 °C, BocNHNH_2_ insertion, deprotection and hydrazone formation in the presence of aldehydes **15a**,**b**.

All synthesized compounds were evaluated for their anticancer activity against lymphoma, hepatocarcinoma, and colon epithelial carcinoma on H9, Huh7 and Caco-2 cells, respectively (Table 2). Healthy FB789 fibroblasts were used as a reference for cytotoxicity determinations. TSIs were calculated as the ratio of CC_50_ values obtained with FB789 cells and, in turn, H9, Huh7 and Caco-2.

In detail, cancer and normal cells were incubated in the presence of an increasing concentration of pyrimidine and pyridine derivatives for 48 h. As reported in Table 2, derivatives **14a**, **14b** and **21b** are characterized by a potency on FB789 of the same order of magnitude of the antineoplastic activity in all cancer cell lines evaluated, as expressed by a TSI < 1 (Table 2, entries 1, 2 and 6). Pyrimidine **21a** had a different behavior, showing moderate selectivity on the Huh7 (TSI = 3.3) and Caco-2 (TSI = 5.0) cell lines, but no selectivity on the H9 cell line (Table 2, entries 5). Similarly, compounds **19a**,**b** displayed high selectivity on the Huh7 (14.0 and 10.2 TSIs for **19a** and **19b**, respectively) and Caco-2 (7.0 and 15.3 TSIs for **19a** and **19b**, respectively) cell lines (Table 2, entries 3 and 4), and low effects on the H9 cell line (0.5 and 1.3 TSIs for **19a** and **19b**, respectively). The difference observed between H9 and Caco-2/Huh7 could be imputable to the different nature and origin of these cell lines, considering that H9 is a suspension cell line derived by lymphoma, while Caco-2 and Huh7 are both adherent epithelial cell lines derived by carcinoma.

Regarding pyridines, derivatives **27a**,**b** showed a low selectivity (TSI < 3) on H9, Huh7 and Caco-2 cells (Table 2, entries 7 and 8). Compounds **33a** and **38a** are characterized by a low potency on FB789 (Table 2, entries 9 and 11). Pyridines **33b** and **38b** instead showed high TSI values in all cells evaluated, due to a very low cytotoxic effect on healthy fibroblasts (Table 2, entries 10 and 12), associated with a potency that ranged from 23 μM in the worst case to 8 μM in the best case.

Comparing novel derivatives with original compounds **1**–**9**, it appears that shifting from a triazine core to a pyrimidine and pyridine nuclei led to an increase in TSI values (Table 1 and Table 2). In particular, comparison of structurally similar derivatives **19a** and **7** highlights an important increase in TSI on Huh7 in the former (14.0 for **19a** and 3.5 for **7**) and a comparable value in Caco-2 (7.0 vs. 8.8). Pyrimidine **19b** and pyridine **33b** show a robust increase in the TSI with respect to the original triazine **9** in all the three cancer cell lines, as TSIs of the latter never exceed 0.5. Compound **21a** has a better TSI of **4** in Huh7 (2.8-fold of difference) and in Caco-2 (1.4-fold). Pyridine **33b** is characterized by 50-, 4.8- and 2.5-fold higher TSIs than triazine **6** on H9, Huh7 and Caco-2 cell lines, respectively.

**Figure 2:**
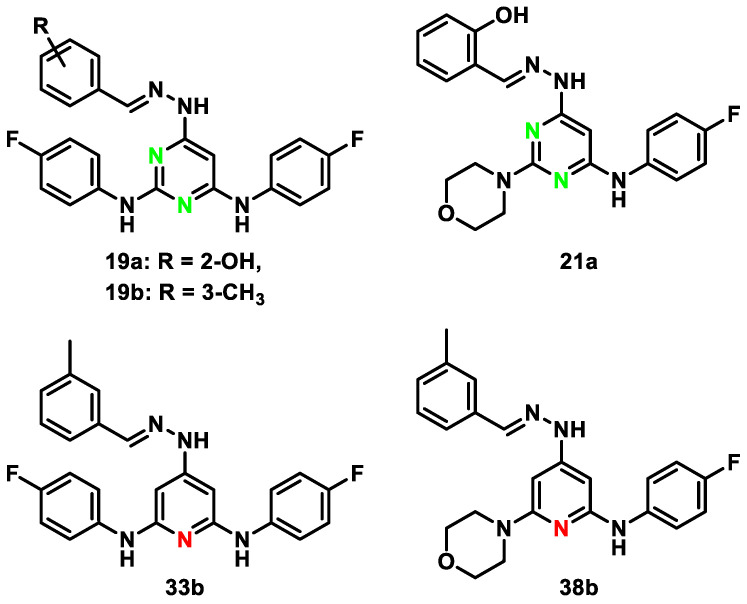
Structure of compounds **19a**, **19b**, **21a**, **33b**, **38b**.

Based on the results obtained using cellular evaluations, further studies aimed at investigating the in vitro ADME properties were conducted either on compounds that showed the highest TSI values, such as **19a**, **19b** and **21a**, or the lowest cytotoxic effect on normal cells, products **33b** and **38b** (Figure 2).

Firstly, the kinetic aqueous solubility of all five derivatives was studied, through the dilution of a DMSO stock solution of these compounds with Mill-Q H_2_O followed by incubation for 3 h at room temperature (RT). As reported in Table 3, all pyrimidine derivatives **19a**, **19b**, and **21a** showed suboptimal values of water solubility, less than 0.1 µg/mL (limit of detection, LOD).

Indeed, the presence of the morpholine and phenol moieties in **21a** did not offer a significant improvement to the water solubility in comparison to **19a** (with a phenol group and two para fluorophenyl motifs) or **19b** (with two para fluorophenyl groups and 3-methylphenyl residue). The replacement of the pyrimidine scaffold with a pyridine one slightly enhances the solubility only when one fluorophenyl group was substituted with a morpholine. In fact, if **38b** showed a LogS value of −5.87, **33b** confirmed the trend seen for **19a**, **19b**, and **21a**. The aqueous solubility of selected compounds was also predicted using the online tool SwissADME; moving from the data obtained for **38b**, the predicted value result was quite similar to the experimental one, suggesting the reliability of the prediction. The predicted values obtained for **19a**, **19b**, **21a**, and **33b** confirmed the suboptimal water solubility of tested derivatives with LogS values between −6.69 and −7.87.

The parallel artificial membrane permeability assay (PAMPA) underlined a general tendency to efficiently cross membranes. As reported in Table 4, the pyrimidine derivatives **19a**, **19b**, and **21a** showed good apparent permeability values, probably due to the scaffold that reduces the aqueous solubility and promotes the interaction with the phospholipid bilayer. Indeed, **19a** and **19b**, both characterized by two para fluorophenyl groups, resulted in good apparent permeability values and higher percentages of membrane retention (20.99% and 27.42%, respectively) than **21a** endowed with a good passive permeability and a very low membrane retention due to the presence of a morpholine moiety (P_app_ 4.80 × 10^−6^ cm/s, MR 25.9%).

Among the pyridine derivatives, the two para fluorophenyl moieties of **33b** significantly increased the membrane retention up to 46.84% when compared to **38b**, whose percentage remained around 20.99%. In terms of apparent permeability, the high interaction of **33b** with the lipidic bilayer influenced its ability to cross the membrane; indeed, the Papp value was noticeably reduced to 1.38 × 10^−6^ cm/s in comparison to **38b** whose P_app_ value was 6.35 × 10^−6^ cm/s.

Regarding the stability of tested compounds in the presence of human liver microsomes, the five derivatives showed percentages of metabolic stability never lower than 97%, suggesting a generally high resistance to the transformations induced by liver microsomes (Table 5). Compounds **19a**, **19b**, and **21a** showed very stable results (>99.9%), while **33b** and **38b** underwent a slight metabolization leading to the formation of oxidized derivative M_1_ (1.68% and 2.06%, respectively). 

Finally, the stability in human plasma was investigated by incubating a DMSO solution of compounds in the presence of HEPES buffer and human plasma for 24 h. According to Table 5, all tested derivatives underwent a slight metabolization, leading to percentages of plasmatic stabilities never lower than 81% after 24 h of incubation.

## 3. Materials and Methods

### 3.1. Chemistry—General Part

All reactions were performed in flame-dried glassware under a nitrogen atmosphere. Reagents were obtained from commercial suppliers (Merck Srl, Milan, Italy) and used without further purification. TLC chromatography was performed on precoated aluminum silica gel SIL G/UV254 plates (Macherey-Nagel & Co., Düren, Deutschland). The detection occurred via fluorescence quenching or development in a ninhydrin solution (0.2 g of ninhydrin in 99.5 mL ethanol and 0.5 mL acetic acid.). Merck silica gel 60 was used for flash chromatography (23–400 mesh). ^1^H NMR and ^13^C NMR spectra were measured on a Bruker (Billerica, MA, USA) Avance DRX400 (400 MHz/100 MHz) spectrometer. Chemical shifts for protons are reported in parts per million (ppm, *δ* scale) and internally referenced to the deuterated dimethyl sulfoxide (DMSO-*d*_6_), methanol (CD_3_OD) or chloroform (CDCl_3_) signal at *δ* 2.50, 3.33 and 7.28 ppm, respectively. ^1^H-NMR spectra are reported first by their multiplicity and then by their number of protons; signals are characterized as: s (singlet), d (doublet), dd (doublet of doublets), ddd (doublet of doublet of doublets), t (triplet), m (multiplet), bs (broad signal). 2,4,6-trichlorotriazine, 2,4,6-trichloropyrimidine and 2,4,6-trifluoropyridine were bought from Merck Srl (Milan, Italy). 

Microwave irradiation experiments were conducted using a CEM Discover Synthesis Unit (CEM Corp., Matthews, NC, USA). The apparatus consists of a continuous focused microwave power delivery system with an operator-selectable power output of 0 to 300 W. The temperature of the contents of the tube was monitored using a calibrated IR temperature control mounted under the reaction tube. All experiments were performed using a stirring option where the contents of the tube are stirred by means of a rotating magnetic plate located below the floor of the microwave cavity and a Teflon-coated magnetic stir bar in the tube.

Mass detention of compounds was performed using an Agilent 1260 Infinity HPLC-DAD system connected to an Agilent MSD 6130 system (Agilent Technologies, Palo Alto, CA, USA) as described in the UV/LC-MS method section below. High-Resolution Mass Spectra (HRMS) were recorded on an LC-MS/MS system (Q Executive Plus; Thermo Scientific, Waltham, MA, USA).

### 3.2. Chemistry—Experimental Procedures and Compound Characterization

The procedure for the synthesis of monosubstituted pyrimidine derivatives 11 and 12 is as follows: 

To a solution of 2,4,6-trichloropyrimidine **10** (1 equiv.) in CH_2_Cl_2_ (10 mL) at 0 °C, morpholine (1 equiv.) was slowly added, followed by DIPEA (1 equiv.). The reaction mixture was stirred at 0 °C for 5 h, and then warmed up to 25 °C. The mixture was washed with H_2_O and brine. The organic phase was dried over anhydrous Na_2_SO_4_ and evaporated to dryness. The resulting residue was purified through column chromatography using a mixture of petroleum ether (PET)/ethyl acetate (EtOAc) 4:1 to give the desired products **11** and **12** with 19% and 68% yield, respectively. 

Compound **11**

R*_f_* = 0.29 (PET/EtOAc 4:1); ^1^H NMR (400 MHz, CDCl_3_) *δ* 6.57 (s, 1H), 3.83 (d, *J* = 5.2 Hz, 4H), 3.76 (d, *J* = 4.8 Hz, 4H) ppm. ^13^C NMR (100 MHz, CDCl_3_) *δ* 161.8, 160.6, 108.3, 66.6, 44.4 ppm. MS *m*/*z* for C_8_H_10_Cl_2_N_3_O, (ESI^+^) *m/z*: 234.0 [M + H]^+^.

Compound **12**

R*_f_* = 0.26 (PET/EtOAc 4:1); ^1^H NMR (400 MHz, CDCl_3_) *δ* 6.41 (s, 1H), 3.79–3.77 (m, 4H), 3.65 (s, 4H) ppm. ^13^C NMR (100 MHz, CDCl_3_) *δ* 163.3, 160.7, 159.9, 99.7, 66.2, 44.6 ppm. MS *m*/*z* for C_8_H_10_Cl_2_N_3_O, (ESI^+^) *m*/*z*: 234.0 [M + H]^+^.

*Procedure for the synthesis of disubstituted pyrimidine derivative* **13**

Compound **12** (1 equiv.) was dissolved in 1,4-dioxane (10 mL) and morpholine (2.5 equiv.) was added. Then, a few drops of HCl were slowly added. The reaction was conducted under microwave irradiation for 40 min at 110 °C. After cooling to 25 °C, the mixture was washed with H_2_O and brine. The organic phase was dried over anhydrous Na_2_SO_4_ and evaporated to dryness. The solid residue obtained was purified using column chromatography using diethyl ether (Et_2_O)/PET 2:1 as eluant to give the disubstituted intermediate **13** with 90% yield.

R*f* = 0.26 (Et_2_O/PET); ^1^H NMR (400 MHz, CDCl_3_) *δ* 5.87 (s, 1H), 3.76–3.73 (m, 8H), 3.56–3.54 (m, 8H) ppm. ^13^C NMR (100 MHz, CDCl_3_) *δ* 163.5, 160.8, 160.6, 91.1, 66.8, 66.5, 44.4, 44.3 ppm. MS *m*/*z* for C_12_H_18_ClN_4_O_2_, (ESI^+^) *m/z*: 285.1 [M + H]^+^.

*General Procedure for the synthesis of trisubstituted pyrimidine derivatives* **14a**,**b**

Compound **13** (1 equiv.) was dissolved in 1,4-dioxane (1 mL) and hydrazine hydrate (20 equiv.) was added. The reaction was stirred under microwave irradiation for 4 h at 100 °C. After this time, the reaction mixture was diluted with EtOAc and then was washed with H_2_O and brine, dried over anhydrous Na_2_SO_4_ and evaporated to dryness. The crude residue was pure enough to be subjected to the next step.

To a solution of hydrazine derivative (1 equiv.) in MeOH (10 mL), the opportune aldehyde (1 equiv.) and a drop of acetic acid were added. The mixture was stirred for 24 h at 25 °C. The precipitate formed was filtered-off and dried under high vacuum giving the desired products **14a**,**b** with 55 and 65% yield, respectively. 

Compound **14a**

R*_f_* = 0.19 (hexane (Hex)/EtOAc 1:1). ^1^H-NMR (400 MHz, CDCl_3_) *δ* 10.70 (bs, 1H), 8.53 (bs,1H), 7.90 (s 1H), 7.31–7.26 (m, 1H), 7.19 (d, *J* = 6.8 Hz, 1H), 7.00 (d, *J* = 8.4 Hz, 1H), 6.93 (t, *J* = 7.2 Hz, 1H), 5.56 (s 1H), 3.80–3.73 (m, 12H), 3.61–3.57 (m, 4H) ppm. ^13^C NMR (100 MHz, CDCl_3_) *δ* 164.1, 160.6, 157.3, 144.0, 130.9, 130.1, 119.7, 118.0, 116.6, 72.9, 66.9, 66.7, 44.6, 44.5 ppm. MS *m*/*z* for C_19_H_25_N_6_O_3_, (ESI^+^) *m*/*z*: 385.2 [M + H]^+^. HRMS (ESI) *m*/*z* for C_19_H_25_N_6_O_3_ = 385.1983 [M + H]^+^, found 385.1985; elemental analysis calcd. (%) for C_19_H_24_N_6_O_3_: C, 59.36; H, 6.29; N, 21.86; O, 12.48; found: C, 59.34; H, 6.29; N, 21.86; O, 12.49.

Compound **14b**

R*_f_* = 0.25 (Hex/EtOAc 1:1). ^1^H NMR (400 MHz, CDCl_3_) *δ* 8.14 (bs, 1H), 7.69 (bs, 1H), 7.48–7.45 (m, 2H), 7.31–7.25 (m, 1H), 7.17 (d, *J* = 7.2 Hz, 1H), 5.92 (s, 1H), 3.82–3.79 (m, 4H), 3.74–3.72 (m, 8H), 3.64–3.62 (m, 4H), 2.40 (s, 3H). ^13^C NMR (100 MHz, CDCl_3_) *δ* 164.3, 162.5, 161.1, 140.5, 138.3, 134.5, 130.1, 128.6, 127.2, 123.9, 73.8, 67.0, 66.8, 44.7, 44.5, 21.4 ppm. MS *m*/*z* for C_20_H_27_N_6_O_2_, (ESI^+^) *m*/*z*: 383.2 [M + H]^+^. HRMS (ESI) *m*/*z* for C_20_H_27_N_6_O_2_ = 383.2190 [M + H]^+^, found 383.2191; elemental analysis calcd. (%) for C_20_H_26_N_6_O_2_: C, 62.81; H, 6.85; N, 21.97; O, 8.37; found: C, 62.80; H, 6.85; N, 21.97; O, 8.38.

*Procedure for the Synthesis of monosubstituted pyrimidine intermediate* **16** *and* **17**

To a solution of 2,4,6-trichloropyrimidine **10** (1 equiv.) in CH_2_Cl_2_ (10 mL) at 0 °C, 4-fluoroaniline (1 equiv.) was slowly added, followed by DIPEA (1 equiv.). The obtained reaction mixture was stirred at 0 °C for 5 h, and then warmed up to 25 °C. The mixture was washed with water, dried over N_2_SO_4_ and the solvent removed under reduced pressure. The crude residue was purified using column chromatography PET/EtOAc 4:1 to give the desired products **16** and **17** with 14 and 75% yield, respectively.

Compound **16**

R*_f_* = 0.29 (PET/EtOAc 4:1). ^1^H NMR (400 MHz, CDCl_3_) δ 7.53–7.50 (m, 2H), 7.09–7.04 (m, 2H), 6.80 (s, 1H) ppm. ^13^C NMR (100 MHz, CDCl_3_) δ 162.0, 159.8 (d, *J* = 149.0 Hz), 158.1, 133.7, 121.9 (d, *J* = 7.9 Hz), 115.8 (d, *J* = 22.5 Hz), 115.7, 111.3 ppm. MS *m*/*z* for C_10_H_7_Cl_2_FN_3_, (ESI^+^) *m*/*z*: 258.0 [M + H]^+^.

Compound **17**

R*_f_* = 0.22 (PET/EtOAc 4:1).^1^H NMR (400 MHz, CDCl_3_) δ 7.30–7.27 (m, 2H), 7.17–7.13 (m, 2H), 6.44 (s, 1H) ppm. ^13^C NMR (100 MHz, CDCl_3_) δ 163.6, 161.8 (d, *J* = 116.0 Hz), 160.2, 160.0, 132.1, 126.4 (d, *J* = 7.1 Hz), 116.9 (d, *J* = 22.7 Hz), 100.7 ppm. MS *m*/*z* for C_10_H_7_Cl_2_FN_3_, (ESI^+^) *m*/*z*: 258.0 [M + H]^+^.

*Procedure for the synthesis of disubstituted pyrimidine intermediate* **18**

Compound **17** was dissolved in 1,4-dioxane (10 mL) and 4-fluoroaniline (2.5 eq) was added. Then, a few drops of HCl were slowly added to the mixture. The reaction was conducted under microwave irradiation for 40 min at 110 °C. The mixture was washed with water, brine and dried over N_2_SO_4_.The residue was purified using column chromatography with CH_2_Cl_2_ as eluant. The purified material was dried in vacuo to afford the desired product with 62% yield.

R*_f_* = 0.20 (CH_2_Cl_2_); ^1^H NMR (400 MHz, CDCl_3_) δ 7.49–7.46 (m, 2H), 7.31–7.27 (m, 2H), 7.11–7.06 (m, 2H), 7.03–6.99 (m, 2H), 6.98 (s, 1H), 6.06 (s, 1H) ppm. ^13^C NMR (100 MHz, CDCl_3_) δ 162.7, 159.9 (d, *J* = 100.1 Hz), 157.6, 134.8, 133.5, 125.4 (d, *J* = 8.0 Hz), 121.8 (d, *J* = 7.7 Hz), 116.3 (d, *J* = 22.6 Hz), 115.5 (d, *J* = 22.3 Hz), 94.4 ppm. MS *m*/*z* for C_16_H_12_ClF_2_N_4_, (ESI^+^) *m*/*z*: 333.1 [M + H]^+^.

*General procedure for the synthesis of trisubstituted pyrimidine derivatives* **19a**,**b**

Compound **18** (1 equiv.) was dissolved in 1,4-dioxane (1 mL) and hydrazine hydrate (20 equiv.) was added. The reaction was stirred under microwave irradiation for 4 h at 100 °C. After this time, the reaction mixture was diluted with EtOAc and then washed with H_2_O and brine, and dried over N_2_SO_4_. The crude residue was pure enough to be subjected to the next step. 

To a solution of hydrazine derivative (1 equiv.) in MeOH (10 mL), the opportune aldehyde (1 equiv.) and a drop acetic acid were added. The mixture was stirred for 48 h at 25 °C. The precipitate that formed was filtered-off and dried under a high vacuum giving the desired products **19a**,**b** with 53 and 61% yield, respectively.

Compound **19a**

R*_f_* = 0.42 (Hex/EtOAc 1:1) ^1^H-NMR (400 MHz, CD_3_OD) δ 8.11 (s, 1H), 7.67 (dd, *J* = 8.8 Hz *J* = 4.4 Hz, 2H), 7.55 (dd, *J* = 8.8 Hz *J* = 4.8 Hz, 2H), 7.29 (d, *J* = 6.4 Hz, 1H), 7.25 (d, 1H), 7.04–7.00 (m, 4H), 6.98–6.90 (m, 2H), 5.76 (s, 1H) ppm. ^13^C NMR (100 MHz, CD_3_OD) δ 162.4, 160.5 (d, *J* = 145.2 Hz), 159.1, 157.1, 156.7, 143.5, 137.0, 136.6, 130.0, 129.4, 122.4 (d, *J* = 7.6 Hz), 120.9 (d, *J* = 7.4 Hz), 119.2, 118.8, 115.9, 114.7 (d, *J* = 22.3 Hz), 114.3 (d, *J* = 22.2 Hz), 76.1 ppm. MS *m*/*z* for C_23_H_19_F_2_N_6_O, (ESI^+^) *m*/*z*: 433.2 [M + H]^+^. HRMS (ESI) *m*/*z* for C_23_H_19_F_2_N_6_O = 433.1583 [M + H]^+^, found 433.1585; elemental analysis calcd. (%) for C_23_H_18_F_2_N_6_O: C, 63.88; H, 4.20; F, 8.79; N, 19.43; O, 3.70; found: C, 63.87; H, 4.20; F, 8.79; N, 19.43; O, 3.72.

Compound **19b**

R*_f_* = 0.30 (Hex/EtOAc 1:1). ^1^H NMR (400 MHz, CD_3_OD) δ 7.88 (s, 1H),7.67–7.64 (m, 2H), 7.57–7.54 (m, 2H), 7.51 (s, 1H), 7.43 (d, *J* = 7.6 Hz, 1H), 7.26(t, *J* = 7.6 Hz, 1H), 7.15 (d, *J* = 7.6, 2H), 7.04–6.95 (m, 4H), 6.09 (s, 1H), 2.36 (s, 3H) ppm. ^13^C NMR (100 MHz, CD_3_OD) δ 162.3, 160.9 (d, *J* = 257.2 Hz), 159.1, 157.2, 156.7, 140.9, 138.0, 135.3, 129.4, 128.1, 126.5, 123.5, 122.3 (d, *J* = 7.5 Hz), 121.0 (d, *J* = 7.4 Hz), 114.6 (d, *J* = 22.3 Hz), 114.3 (d, *J* = 22.2 Hz), 76.9, 20.0 ppm. MS *m*/*z* for C_24_H_21_F_2_N_6_, (ESI^+^) *m*/*z*: 431.2 [M + H]^+^. HRMS (ESI) *m*/*z* for C_24_H_21_F_2_N_6_ = 431.1790 [M + H]^+^, found 431.1792; elemental analysis calcd. (%) for C_24_H_20_F_2_N_6_: C, 66.97; H, 4.68; F, 8.83; N, 19.52; found: C, 66.95; H, 4.69; F, 8.83; N, 19.53.

*Procedure for the synthesis of disubstituted pyrimidine* **20**

To a solution of compound **17** (1 equiv.) in EtOH (10 mL), morpholine (1 equiv.) and triethyl amine (1.5 equiv.) were added. The reaction mixture was stirred at 80 °C for 18 h. After this time, the solvent was evaporated under reduced pressure and the residue was washed with water and brine, and dried over Na_2_SO_4_. The obtained crude was purified using column chromatography using CH_2_Cl_2_/MeOH (10 mL:100 μL) as eluant. The purified material was dried in vacuo to afford the desired product with 62% yield.

R*_f_* = 0.20 (CH_2_Cl_2_/MeOH 10mL:100 μL). ^1^H NMR (400 MHz, CDCl_3_) δ 7.29–7.26 (m, 2H), 7.08–7.04 (m, 2H), 6.55 (s,1H), 5.89 (s, 1H), 3.75 (d, *J* = 4 Hz, 8H) ppm. ^13^C NMR (100 MHz, CDCl_3_) δ 164.3, 161.6 (d, *J* = 200.2 Hz), 158.0, 135.5, 124.2 (d, *J* = 7.1 Hz), 115.9 (d, *J* = 22.4 Hz), 73.9, 67.0, 66.6, 44.6, 44.5 ppm. MS *m*/*z* for C_14_H_15_ClFN_4_O, (ESI^+^) *m*/*z*: 309.1 [M + H]^+^.

*Procedure for the synthesis of disubstituted pyrimidine* **21a**,**b**

Compound **20** (1 equiv.) was dissolved in 1,4-dioxane (1 mL) and hydrazine hydrate (20 equiv.) was added. The reaction was stirred under microwave irradiation for 6 h at 100 °C. After this time, the reaction mixture was diluted with EtOAc and then washed with H_2_O and brine, and dried over N_2_SO_4_. The crude residue was pure enough to be subjected to the next step.

To a solution of hydrazine derivative (1 equiv.) in MeOH (10 mL), the opportune aldehyde (1 equiv.) and a drop acetic acid were added. The mixture was stirred for 48 h at 25 °C. The precipitate formed was filtered-off and dried under high vacuum giving the desired products **21a**,**b** with 46% and 45% yield, respectively.

Compound **21a**

R*_f_* = 0.18 (CH_2_Cl_2_/MeOH 10:0,2). ^1^H-NMR (400 MHz, CDCl_3_) δ 10.48 (bs, 1H), 8.08 (bs, 1H), 7.88 (s, 1H), 7.36–7.33 (m, 2 H), 7.29–7.26 (m, 1H), 7.18 (dd, *J* = 7.6 Hz, *J* = 1.6 Hz, 1H), 7.08 (t, *J* = 8.8 Hz, 2 H), 7.00 (d, *J* = 7.6 Hz, 1H), 6.92 (t, *J* = 8.4 Hz, 1H), 6.45 (bs, 1H), 5.67 (s, 1H), 3.76 (s, 8H) ppm. ^13^C-NMR (100 MHz, CDCl_3_) δ 162.3, 161.0, 159.3 (d, *J* = 251.4 Hz), 157.4, 144.2, 135.0, 131.0, 130.1, 123.6 (d, *J* = 8.0 Hz), 119.6, 117.9, 116.8, 115.9 (d, *J* = 22.4 Hz), 74.6, 66.9, 44.6 ppm. MS *m*/*z* for C_21_H_22_FN_6_O_2_, (ESI^+^) *m*/*z*: 409.2 [M + H]^+^. HRMS (ESI) *m/z* for C_21_H_22_FN_6_O_2_ = 409.1783 [M+H]^+^, found 409.1781; elemental analysis calcd. (%) for C_21_H_21_FN_6_O_2_: C, 61.76; H, 5.18; F, 4.65; N, 20.58; O, 7.83; found: C, 61.75; H, 5.19; F, 4.65; N, 20.58; O, 7.84.

Compound **21b**

R*_f_*: 0.29 (CH_2_Cl_2_/MeOH 10:0,2) ^1^H NMR (400 MHz, CDCl_3_) δ 8.10 (bs, 1H), 7.69 (s, 1H), 7.44 (s, 1H), 7.41–7.35 (m, 3H), 7.29–7.25 (m, 1H), 7.18 (d, *J* = 7.6 Hz, 1H), 7.09–7.04 (m, 2H), 6.39 (bs, 1H), 6.03 (s, 1H), 3.76 (s, 8H), 2.38 (3H) ppm. ^13^C NMR (100 MHz, DMSO-*d_6_*) δ 162.4, 161.8, 160.0 (d, *J* = 309.8 Hz), 156.1, 140.8, 138.4, 138.0, 135.6, 130.0, 129.1, 126.8, 123.9, 121.1 (d, *J* = 7.4 Hz), 115.5 (d, *J* = 21.8 Hz), 76.5, 66.6, 44.7, 21.5 ppm. MS *m*/*z* for C_22_H_24_FN_6_O, (ESI^+^) *m*/*z*: 407.2 [M + H]^+^. HRMS (ESI) *m/z* for C_22_H_24_FN_6_O = 407.1990 [M + H]^+^, found 407.1991; elemental analysis calcd. (%) for C_22_H_23_FN_6_O: C, 65.01; H, 5.70; F, 4.67; N, 20.68; O, 3.94; found: C, 65.00; H, 5.71; F, 4.67; N, 20.68; O, 3.95.

*Procedure for the synthesis of monosubstituted pyridine derivatives* **23** *and* **24**

To a solution of morpholine (1 equiv.) and Et_3_N (2.5 equiv.) in dry THF (5 mL), a solution of 2,4,6-trichlorpyridine **22** (1 equiv.) in THF (2 mL) was added. The reaction mixture was stirred 24 h at 65 °C. After cooling to 25 °C, the reaction was concentrated to remove THF and then diluted with Et_2_O and washed with water and brine, dried over anhydrous Na_2_SO_4_, and filtered and evaporated in vacuo. The crude mixture was purified via column chromatography using Hex/EtOAc 5:1 as the eluant to give **23** and **24** with 15 and 30% yield, respectively.

Compound **23**

R*_f_* = 0.17 (Hex/EtOAc 5:1). ^1^H NMR (400 MHz, CDCl_3_) δ 6.57 (s, 2H), 3.81 (t, *J* = 4 Hz, 4H), 3.30 (t, *J* = 4 Hz, 4H) ppm. ^13^C NMR (100 MHz, CDCl_3_) δ 158.0, 151.2, 106.2, 66.0, 46.1 ppm. MS *m*/*z* for C_9_H_11_Cl_2_N_2_O, (ESI^+^) *m*/*z*: 233.0 [M + H]^+^.

Compound **24**

R*_f_* = 0.40 (Hex/EtOAc 5:1). ^1^H NMR (400 MHz, CDCl_3_) δ = 6.66 (d, *J* = 1.2 Hz, 1H), 6.47 (d, *J* = 4 Hz, 1H), 3.79 (t, *J* = 4.8 Hz, 4H), 3.53 (t, *J* = 4 Hz, 4H) ppm. ^13^C NMR (100 MHz, CDCl_3_) δ 159.2, 150.2, 146.3, 112.6, 104.4, 66.4, 45.1 ppm. MS *m*/*z* for C_9_H_11_Cl_2_N_2_O, (ESI^+^) *m*/*z*: 233.0 [M + H]^+^.

*Procedure for the Synthesis of disubstituted pyridine derivatives* **25** *and* **28**

To a solution of monosubstituted pyridine **24** (1 equiv.) in DMF (2 mL), morpholine (10 equiv.) and DIPEA (3 equiv.) was added. The mixture was warmed at 120 °C for 48 h. After cooling to 25 °C, the reaction mixture was diluted with CH_2_Cl_2_ and washed with an aqueous solution of 3% LiCl. The organic phase was dried over anhydrous Na_2_SO_4_, and filtered and evaporated in vacuo to afford a crude mixture of two regioisomers. Products **25** and **28** can be separated through column chromatography using a mixture of Hex/EtOAc 2:1, and recovered with 58 and 18% yield, respectively.

Compound **25**

R*_f_* = 0.45 (Hex/EtOAc 2:1)] ^1^H NMR (400 MHz, CDCl_3_) δ 6.0 (s, 2H), 3.79 (d, *J* = 4 Hz, 8H), 3.47 (d, *J* = 8 Hz, 8H) ppm. ^13^C NMR (100 MHz, CDCl_3_) δ 158.2, 146.8, 97.0, 66.7, 49.3 ppm. MS *m*/*z* for C_13_H_19_ClN_3_O_2_, (ESI^+^) *m*/*z*: 284.1 [M + H]^+^.

Compound **28**

R*_f_* = 0.13 (Hex/EtOAc 2:1). ^1^H NMR (400 MHz, CDCl_3_) δ 6.20 (d, *J* = 4 Hz, 1H), 5.77 (d, *J* = 1,2 Hz, 1H), 3.82–3.78 (m, 8H), 3.46 (t, *J* = 4 Hz, 4H), 3.25 (t, *J* = 4 Hz, 4H) ppm. ^13^C NMR (100 MHz, CDCl_3_) δ 159.2, 150.2, 146.3, 112.6, 104.4, 66.4, 45.1 ppm. MS *m*/*z* for C_13_H_19_ClN_3_O_2_, (ESI^+^) *m*/*z*: 284.1 [M + H]^+^.

*Procedure for the synthesis of Boc-protected trisubstituted pyridine carbazate* **26**

In a microwave tube, compound **25** (1 equiv.), *tert*-butyl carbazate (BocNHNH_2_, 10 equiv.), Pd_2_(dba)_3_ (0.04 equiv.), DPPF (0.12 equiv.), Cs_2_CO_3_ (2 equiv.) and dry toluene (1 mL) were added under an argon atmosphere. The reaction mixture was stirred at 150 °C for 10 min under microwave irradiation. The mixture was filtered through celite, rinsed with EtOAc and concentrated. The crude was purified using column chromatography with CH_2_Cl_2_/EtOAc 4:1 as the eluant to produce the product **26** with 65% yield.

R*_f_* = 0.43 (Hex/EtOAc 2:1)] ^1^H NMR (400 MHz, CDCl_3_) δ 6.2 (s, 2H), 3.98–3.85 (m, 8H), 3.81 (t, *J* = 4.8 Hz, 4H), 3.50 (t, *J* = 4.4 Hz, 4H), 1.49 (s, 3H) ppm. ^13^C NMR (100 MHz, CDCl_3_) δ 159.0, 154.4, 153.2, 89.9, 82.6, 66.9, 46.0, 28.3 ppm. MS *m*/*z* for C_18_H_30_N_5_O_4_, (ESI^+^) *m*/*z*: 380.2 [M + H]^+^.

*Procedure for the synthesis of trisubstituted pyridine derivatives* **27a**,**b**

Boc-protected pyridine **26** (1 equiv.) was dissolved in anhydrous CH_2_Cl_2_ (430 µL) and cooled to 0 °C. TFA (290 µL) was added, and the reaction mixture was warmed to 25 °C and stirred for 1 h. After this time, the mixture was concentrated at reduced pressure. The residue obtained was pure enough to be subjected to the next step.

To a solution of hydrazine derivative (1 equiv.) in MeOH (10 mL), the opportune aldehyde (1 equiv.) and few drops of acetic acid were added. The mixture was stirred for 24 h at 25 °C and concentrated under vacuum. The residue was purified using column chromatography (Hex/EtOAc 2:1), furnishing the desired products **27a**,**b** with 39% and 18% yield, respectively.

Compound **27a**

R*_f_* = 0.40 (Hex/EtOAc 1:1). ^1^H NMR (400 MHz, CDCl_3_) δ 10.93 (s, 1H), 8.39 (s, 1H), 7.86 (s, 1H), 7.27–7.24 (m, 1H), 7.15 (dd, *J* = 7.6 Hz, *J* = 1.6 Hz, 1H), 6.99 (d, *J* = 8.0 Hz, 1H), 6.92–6.90 (m, 1H), 5.96 (m, 1H), 3.85–3.78 (m, 8H), 3.44 (t, *J* = 4.0 Hz, 4H), 3.31 (t, *J* = 4.0 Hz, 4H) ppm. ^13^C NMR (CDCl_3_, 100 Hz) δ 160.2, 159.6, 157.1, 155.3, 141.7, 130.2, 129.5, 119.6, 118.5, 116.5, 82.1, 66.8, 66.6, 47.0, 46.2 ppm. MS *m*/*z* for C_20_H_26_N_5_O_3_, (ESI^+^) *m*/*z*: 384.2 [M + H]^+^. HRMS (ESI) *m*/*z* for C_20_H_26_N_5_O_3_ = 384.2030 [M + H]^+^, found 384.2031; elemental analysis calcd. (%) for C_20_H_25_N_5_O_3_: C, 62.65; H, 6.57; N, 18.26; O, 12.52; found: C, 62.64; H, 6.58; N, 18.26; O, 12.53.

Compound **27b**

R*_f_* = 0.55 (Hex/EtOAc 2:1). ^1^H NMR (400 MHz, CDCl_3_) δ = 8.11 (s, 1H), 7.66 (s, 1H), 7.47 (d, *J* = 8.0 Hz, 2H), 7.27 (t, *J* = 8.0 Hz, 1H), 7.14 (d, *J* = 8.0 Hz, 1H), 6.33–6.32 (m, 1H), 3.86–3.80 (m, 8H), 3.42 (t, *J* = 8.0 Hz, 4H), 3.33 (t, *J* = 8.0 Hz, 4H), 2.39 (s, 3H) ppm. ^13^CNMR (CDCl_3_, 100 Hz) δ 163.3, 159.4, 153.8, 141.8, 146.2, 138.3, 134.2, 130.7, 128.5, 127.9, 124.1, 81.2, 66.2, 66.1, 46.3, 21.4 ppm. MS *m*/*z* for C_21_H_28_N_5_O_2_, (ESI^+^) *m*/*z*: 382.2 [M+H]^+^. HRMS (ESI) *m*/*z* for C_21_H_28_N_5_O_2_ = 382.2238 [M + H]^+^, found 382.2239; elemental analysis calcd. (%) for C_21_H_27_N_5_O_2_: C, 66.12; H, 7.13; N, 18.36; O, 8.39; found: C, 66.13; H, 7.14; N, 18.36; O, 8.37.

*Procedure for the synthesis of monosubstituted pyridine derivatives* **29** *and* **30**

In a two necks flask equipped with molecular sieves, 2,4,6-tricloropyridine **22** (1 equiv.), Cs_2_CO_3_ (1 equiv.), Pd(PPh_3_)_4_ (0.02 equiv.) and dry toluene (5 mL) were added. Subsequently, 4-fluoroaniline (1.6 equiv.) was added under an argon atmosphere and the reaction mixture was stirred for 24 h at 150 °C. After this time, the mixture was filtered through celite, rinsed with EtOAc and concentrated. The residue obtained was diluted with EtOAc, washed with a saturated aqueous solution of NaHCO_3_ and brine, dried over Na_2_SO_4_ and concentrated under reduced pressure. The crude was purified using column chromatography with Pet/EtOAc 4:1 as eluant to give the products **29** and **30** with 19 and 50% yield, respectively.

Compound **29**

R*_f_* = 0.50 (Pet/EtOAc 4:1). ^1^H NMR (400 MHz, CDCl_3_) δ 7.27 (m, 2H), 7.12 (t, *J* = 8.4 Hz, 2H), 7.00 (s, 1H), 6.7 (s, 2H) ppm. ^13^C (100 MHz, CDCl_3_) δ 159.3 (d, *J* = 241.9 Hz), 156.2, 146.3, 135.6, 123.8 (d, *J* = 6.9 Hz), 116.0 (d, *J* = 22.3 Hz), 98.0 ppm. MS *m*/*z* for C_11_H_8_Cl_2_FN_2_, (ESI^+^) *m*/*z*: 257.0 [M + H]^+^.

Compound **30**

R*_f_* = 0.65 (Pet/EtOAc 4:1). ^1^H NMR (400 MHz, CDCl_3_) δ 7.26 (m, 2H), 7.09 (t, *J* = 8.8 Hz, 2H), 7.00 (s, 1H), 6.73 (d, *J* = 1.2 Hz, 1H), 6.54 (d, *J* = 1.2 Hz, 1H) ppm. ^13^C NMR (100 MHz, CDCl_3_) δ 160.0 (d, *J* = 240.3 Hz), 157.2, 150.5, 146.6, 134.5, 124.8 (d, *J* = 8.1 Hz), 116.4 (d, *J* = 22.6 Hz), 114.2, 104.9 ppm. MS *m*/*z* for C_11_H_8_Cl_2_FN_2_, (ESI^+^) *m*/*z*: 257.0 [M + H]^+^.

*Procedure for the synthesis of disubstituted pyridine derivatives* **31** *and* **34**

Pd(OAc)_2_ (0.02 equiv.) and BINAP (0.02 equiv.) were dissolved in anhydrous toluene (1 mL) and the mixture was kept under argon for 10 min. At the same time, compound **30** (1 equiv.), anhydrous toluene (1 mL), Cs_2_CO_3_ (3.5 equiv.), and 4-fluoroaniline (1.5 equiv.) and then the solution of Pd(OAc)_2_ and BINAP above described were put into a Schenck tube. The reaction mixture was warmed at 160 °C and left under stirring for 4 h. After this time, the mixture was filtered through celite, rinsed with EtOAc and concentrated. The crude was purified using column chromatography with Pet/EtOAc 3:1 as eluant to give the products **31** and **34** with 36 and 13% yield, respectively.

Compound **31**

R*_f_* = 0.63 (Pet/EtOAc 3:1). ^1^H NMR (400 MHz, CDCl_3_) δ 7.29 (m, 4H), 7.06 (m, 4H), 6.65 (s, 1H), 6.13 (s, 2H) ppm. ^13^C NMR (100 MHz, CDCl_3_) δ 159.2 (d, *J* = 260.2 Hz), 156.0, 146.6, 135.4, 123.9 (d, *J* = 7.9 Hz), 116.1 (d, *J* = 22.4 Hz), 98.0 ppm. MS *m*/*z* for C_17_H_13_ClF_2_N_3_, (ESI^+^) *m/z*: 332.1 [M + H]^+^.

Compound **34**

R*_f_* = 0.44 (Pet/EtOAc 3:1). ^1^H NMR (400 MHz, CDCl_3_) δ 7.21 (m, 4H), 7.11 (m, 4H), 6.45 (s, 1H), 6.24 (d, *J* = 1.6 Hz, 1H), 6.0 (d, *J* = 1.6 Hz, 1H), 5.9 (s, 1H) ppm. ^13^C (100 MHz, CDCl_3_) δ 159.2 (d, *J* = 270.4 Hz), 156.8, 148.1, 141.5, 136.5, 123.4 (d, *J* = 7.4 Hz), 116.3 (d, *J* = 22.4 Hz), 100.0, 94.0 ppm. MS *m*/*z* for C_17_H_13_ClF_2_N_3_, (ESI^+^) *m*/*z*: 332.1 [M + H]^+^.

*Procedure for the synthesis of Boc-protected trisubstituted pyridine carbazate* **32**

In a microwave tube, compound **31** (1 equiv.), BocNHNH_2_ (10 equiv.), Pd_2_(dba)_3_ (0.04 equiv.), DPPF (0.12 equiv.), Cs_2_CO_3_ (2 equiv.) and dry toluene (1 mL) were added under an argon atmosphere. The reaction mixture was stirred at 150 °C for 10 min under microwave irradiation. The mixture was filtered through celite, rinsed with EtOAc and concentrated. The crude was purified using column chromatography with Hex/EtOAc 2:1 as eluant to produce the product **32** with 59% yield.

R*_f_* = 0.37 (Hex/EtOAc 2:1). ^1^H NMR (400 MHz, CDCl_3_): δ 7.31 (m, 4H), 7.03 (m, 4H), 6.61 (s, 2H), 5.32 (s, 1H), 4.24 (s, 1H), 1.49 (s, 3H) ppm. ^13^C NMR (100 MHz, CDCl_3_) δ 158.8 (d, *J* = 240.2 Hz), 155.6, 154.2, 153.3, 136.5, 123.0 (d, *J* = 7.7 Hz), 115. (d, *J* = 22.2 Hz), 90.9, 83.1, 28.2 ppm. MS *m*/*z* for C_22_H_24_F_2_N_5_O_2_, (ESI^+^) *m/z*: 428.2 [M + H]^+^.

*Procedure for the synthesis of trisubstituted pyridine derivativities* **33a**,**b**

Boc-protected pyridine **32** (1 equiv.) was dissolved in anhydrous CH_2_Cl_2_ (430 µL) and cooled to 0 °C. TFA (290 µL) was added, and the reaction mixture was warmed to 25 °C and stirred for 1 h. After this time, the mixture was concentrated at reduced pressure. The residue obtained was pure enough to be subjected to the next step.

To a solution of hydrazine derivative (1 equiv.) in MeOH (10 mL), the opportune aldehyde (1 equiv.) and few drops of acetic acid were added. The mixture was stirred for 24 h at 25 °C and concentrated under vacuum. The residue was purified using column chromatography (Hex/EtOAc 1:1) furnishing the desired products **33a**,**b** with 29% and 21% yield, respectively.

Compound **33a**

R*_f_* = 0.37 (Hex/EtOAc 1:1). ^1^H NMR (400 MHz, CDCl_3_) δ 10.47 (bs, 1H), 7.81 (s, 1H), 7.57 (bs, 1H), 7.33–7.27 (m, 5H), 7.15 (d, *J* = 7.6 Hz, 2H), 7.07–6.98 (m, 5H), 6.92 (t, *J* = 7.6 Hz,1H), 6.36 (bs, 2H), 5.81 (s, 2H) ppm. ^13^C (100 MHz, CD_3_OD) δ 161.6, 159.4 (d, *J* = 69.8 Hz), 159.3, 157.4, 156.0, 140.7, 139.4, 136.9, 136.6, 131.0, 126.7, 122.5 (d, *J* = 10.9 Hz), 121.2, 119.6, 114.7 (d, *J* = 19.6 Hz), 114.4 (d, *J* = 19.6 Hz), 81.1 ppm. MS *m*/*z* for C_24_H_20_F_2_N_5_O, (ESI^+^) *m*/*z*: 432.2 [M + H]^+^. HRMS (ESI) *m*/*z* for C_24_H_20_F_2_N_5_O = 432.1630 [M + H]^+^, found 432.1632; elemental analysis calcd. (%) for C_24_H_19_F_2_N_5_O: C, 66.81; H, 4.44; F, 8.81; N, 16.23; O, 3.71; found: C, 66.82; H, 4.45; F, 8.81; N, 16.23; O, 3.70.

Compound **33b**

R*_f_* = 0.49 (Hex/EtOAc 2:1). ^1^H NMR (400 MHz, CDCl_3_) δ 7.72–7.68 (m, 1H), 7.63 (s, 1H), 7.47–7.43 (m, 1H), 7.39–7.37 (m, 1H), 7.31–7.26 (m, 4H), 7.18–7.16 (m, 2H), 7.06–7.02 (m, 4H), 6.43 (bs, 1H), 5.99 (s, 2H), 2.39 (s, 3H) ppm. ^13^C (100 MHz, CDCl_3_) δ 160.3, 156.7 (d, *J* = 226.9 Hz), 154.0, 140.1, 138.4, 136.2, 130.2, 128.6, 127.6, 127.1, 123.9, 123.5 (*d*, *J* = 6.8 Hz), 115.8 (d, *J* = 22.4 Hz), 82.5, 21.3 ppm. MS *m*/*z* for C_25_H_22_F_2_N_5_, (ESI^+^) *m*/*z*: 430.2 [M+H]^+^. HRMS (ESI) *m*/*z* for C_25_H_22_F_2_N_5_ = 430.1838 [M + H]^+^, found 430.1839; elemental analysis calcd. (%) for C_25_H_21_F_2_N_5_: C, 69.92; H, 4.93; F, 8.85; N, 16.31; found: C, 69.93; H, 4.94; F, 8.85; N, 16.31.

*Procedure for the synthesis of disubstituted pyridine derivatives* **35** *and* **36**

In a microwave tube equipped with a stir bar, the monosubstituted pyridine **30** (1 equiv.) dissolved in anhydrous 1,4-dioxane (1.5 mL), morpholine (5 equiv.) and DIPEA (1.5 equiv.) were added. The reaction mixture was irradiated at 160 °C for 10h in a microwave oven. Then, the mixture was extracted with EtOAc, washed with brine, dried over anhydrous Na_2_SO_4_ and concentrated under vacuum. The crude was purified through column chromatography using PET/EtOAc 5:1 as eluant to give products **35** and **36** with 19 and 46% yield, respectively.

Compound **35**

R*_f_* = 0.08 (PET/EtOAc 5:1). ^1^H NMR (400 MHz, CDCl_3_) δ = 7.21 (m, 2H), 7.11 (m, 2H), 6.24 (d, *J* = 1.6 Hz, 1H), 6.0 (d, *J* = 1.6 Hz, 1H), 5.9 (s, 1H), 3.82–3.78 (m, 4H), 3.46 (t, *J* = 4 Hz, 2H), 3.25 (t, *J* = 4 Hz, 2H) ppm. ^13^C (100 MHz, CDCl_3_) δ 161.1, 157.6 (d, *J* = 197.8 Hz), 148.0, 134.8, 124.9 (d, *J* = 6.6 Hz), 124.6, 116.4 (d, *J* = 22.6 Hz), 100.0, 87.3, 68.8, 46.4 ppm. MS *m*/*z* for C_15_H_16_ClFN_3_O, (ESI^+^) *m*/*z*: 308.1 [M + H]^+^.

Compound **36**

R*_f_* = 0.37 (PET/EtOAc 5:1). ^1^H NMR (400 MHz, CDCl_3_) δ 7.27 (m, 2H), 7.05 (t, *J* = 8.8 Hz, 2H), 6.24 (d, *J* = 9.2 Hz, 1H), 6.08 (d, *J* = 4.8 Hz, 1H), 3.81 (t, *J* = 4.8 Hz, 2H), 3.49 (t, *J* = 4Hz, 2H) ppm. ^13^C NMR (100 MHz, CDCl_3_) δ 159.3, 159.1 (d, *J* = 240.2 Hz), 155.7, 146.4, 135.9, 123.4 (d, *J* = 6.7 Hz), 115.9 (d, *J* = 22.4 Hz), 97.4, 96.9, 66.7, 45.4 ppm. MS *m*/*z* for C_15_H_16_ClFN_3_O, (ESI^+^) *m*/*z*: 308.1 [M + H]^+^.

*Procedure for the synthesis of Boc-protected trisubstituted pyridine carbazate* **37**

In a microwave tube, compound **36** (1 equiv.), BocNHNH_2_ (10 equiv.), Pd_2_(dba)_3_ (0.04 equiv.), DPPF (0.12 equiv.), Cs_2_CO_3_ (2 equiv.) and dry toluene (1 mL) were added under an argon atmosphere. The reaction mixture was stirred at 150 °C for 10 min under microwave irradiation. The mixture was filtered through celite, rinsed with EtOAc and concentrated. The crude was purified using column chromatography with Hex/EtOAc 2:1 as eluant to give the product **37** with 42% yield.

R*_f_* = 0.38 (Hex/EtOAc 2:1). ^1^H NMR (400 MHz, CDCl_3_) δ 7.31 (m, 2H), 7.0 (t, *J* = 8.4 Hz, 2H), 6.54 (s, 1H), 6.24 (s, 1H), 3.84 (t, *J* = 4.8 Hz, 2H), 3.51 (t, *J* = 4.8 Hz, 2H) ppm. ^13^C (100 MHz, CDCl_3_) δ 160.3, 159.6 (d, *J* = 240.2 Hz), 155.7, 154.2, 146.2, 135.8, 123.4 (d, *J* = 6.8 Hz), 115.9 (d, *J* = 22.2 Hz), 97.4, 96.8, 80.5, 66.9, 45.9, 28.3 ppm. MS *m*/*z* for C_20_H_27_FN_5_O_3_, (ESI^+^) *m*/*z*: 404.2 [M + H]^+^.

*Procedure for the synthesis of trisubstituted pyridine derivativities* **38a**,**b**

Boc-protected pyridine **37** (1 equiv.) was dissolved in anhydrous CH_2_Cl_2_ (430 µL) and cooled to 0 °C. TFA (290 µL) was added, and the reaction mixture was warmed to 25 °C and stirred for 1h. After this time, the mixture was concentrated at reduced pressure. The residue obtained was pure enough to be subjected to the next step.

To a solution of hydrazine derivative (1 equiv.) in MeOH (10 mL), the opportune aldehyde (1 equiv.) and few drops of acetic acid were added. The mixture was stirred for 24 h at 25 °C and concentrated under vacuum. The residue was purified using column chromatography (Hex/EtOAc 1:1) furnishing the desired products **38a**,**b** with 37% and 33% yield, respectively.

Compound **38a**

R*_f_* = 0.31 (Hex/EtOAc 1:1). ^1^H NMR (400 MHz, CDCl_3_). δ 10.63 (bs, 1H), 7.84 (s, 1H), 7.61 (bs, 1H), 7.46–7.36 (m, 2H), 7.30–7.27 (m, 3H), 7.17 (d, *J* = 7.6 Hz, 1H), 7.06–7.00 (m, 3H), 6. 93 (t, d, *J* = 7.6 Hz 1H), 5.74–5.72 (m, 1H), 3.84–3.81 (m, 4H), 3.50–3.48 (m, 4H) ppm. ^13^C (100 MHz, CDCl_3_) δ 160.3, 156.7 (d, *J* = 113.7 Hz), 152.4, 142.5, 136.7, 130.7, 129.7, 125.0, 122.9, 122.8 (d, *J* = 7.5 Hz), 119.6, 118.0, 116.7, 116.4, 115.7 (d, *J* = 22.3 Hz), 81.8, 66.8, 45.7 ppm. MS *m*/*z* for C_22_H_23_FN_5_O_2_, (ESI^+^) *m*/*z*: 408.2 [M + H]^+^. HRMS (ESI) *m*/*z* for C_22_H_23_FN_5_O_2_ = 408.1830 [M + H]^+^, found 408.1831; elemental analysis calcd. (%) for C_22_H_22_FN_5_O_2_: C, 64.85; H, 5.44; F, 4.66; N, 17.19; O, 7.85; found: C, 64.83; H, 5.45; F, 4.66; N, 17.19; O, 7.86.

Compound **38b**

R*_f_* = 0.43 (Hex/EtOAc 1:1)]. ^1^H NMR (400 MHz, CDCl_3_) δ 7.71 (bs, 1H), 7.65 (s, 1H), 7.44 (d, *J* = 8.8 Hz, 2H), 7–32-7.27 (m, 4H), 7.17 (d, *J* = 7.6 Hz, 1H), 7.05–7.01 (m, 2H), 5.91 (s, 2H), 3.85 (t, *J* = 4.8 Hz, 2H), 3.51 (t, *J* = 4.8 Hz, 2H), 2.42 (s, 3H) ppm. ^13^C (100 MHz, CDCl_3_) δ 162.5, 160.4, 158.1, 154.5, 138.4, 134.2, 130.3, 128.6, 127.3, 124.0, 123.9, 115.8 (d, *J* = 22.3 Hz), 81.9, 66.6, 46.4, 21.3 ppm. MS *m*/*z* for C_23_H_25_FN_5_O, (ESI^+^) *m*/*z*: 406.2 [M + H]^+^. HRMS (ESI) *m*/*z* for C_23_H_25_FN_5_O = 406.2038 [M + H]^+^, found 406.2036; elemental analysis calcd. (%) for C_23_H_24_FN_5_O: C, 68.13; H, 5.97; F, 4.69; N, 17.27; O, 3.95; found: C, 68.11; H, 5.97; F, 4.69; N, 17.27; O, 3.96.

### 3.3. Biology—General Part

All solvents, reagents and human plasma were purchased from Sigma-Aldrich Srl (Milan, Italy). Milli-Q quality water (Millipore, Milford, MA. USA), acetonitrile (ACN) and formic acid (FA) were used for the chromatographic analyses. Cell culture mediums, fetal bovine serum (FBS), L-glutamine, and penicillin–streptomycin were purchased from Euroclone S.p.A. (Milan, Italy). SOF (MCE^®^ cat. HY-15005), REM (MCE^®^ cat. HY-104077) and RAL (MCE^®^ cat. HY-10353), used as reference compounds, were purchased from MedChem Express (https://www.medchemexpress.com, accessed on 20 March 2024). RAL was dissolved in molecular grade water, and SOF and REM were dissolved in 100% dimethyl sulfoxide (DMSO).

### 3.4. Biology—Cells

#### 3.4.1. Cell Culture

Cell-based assays were carried out on the normal human fibroblast FB789 cell line (kindly provided by Elena Dell’Ambra from IRCCS Istituto Dermopatico dell’Immacolata, Rome), on the Huh7 hepatocarcinoma cell line (kindly provided by Istituto Toscano Tumori, Core Research Laboratory, Siena, Italy), on the Caco-2 adenocarcinoma colorectal cell line (ATCC catalog. n. HTB-37), on the suspension H9 cell line (repository code ARP0001, NIBSC Centre for AIDS reagents) and on the adherent TZM-bl cell line (repository code ARP5011, NIBSC Centre for AIDS reagents).

The FB789 was cultured in Dulbecco’s Modified Eagle Medium (DMEM) and Ham’s F10 in a ratio of 50%, supplemented with 10% FBS, 2 mM L-glutamine, and 10,000 unit/mL penicillin/streptomycin.

Huh7 and Caco-2 were used to determine the cytotoxicity and the antiviral activity of candidate compounds against flaviviruses and SARS-CoV-2, respectively. H9 cells in combination with TZM-bl cells were used to evaluate the compounds against HIV-1, as described in the antiviral assays section.

High glucose Dulbecco’s Modified Eagle Medium with sodium pyruvate and L-glutamine (DMEM; Euroclone) was used to grow Huh-7 and TZM-bl. Minimum Essential Eagle Medium (EMEM; Euroclone) was used to propagate Caco-2. DMEM and EMEM were supplemented with 10% fetal bovine serum (FBS; Euroclone) and 1% penicillin/streptomycin (Pen/Strep, Euroclone). The growth medium with a lower concentration of FBS (1%) was used for viral propagation, cytotoxic and antiviral experiments in adherent cell lines. Suspension cell lines were grown, propagated and infected in RPMI 1640 medium supplemented with 10% fetal bovine serum (FBS; Euroclone), 2 mM L-glutamine and 1% penicillin/streptomycin (Pen/Strep, Euroclone). All cell lines were grown at 37 °C in a 5% CO_2_ atmosphere in a humidified incubator.

#### 3.4.2. Cytotoxicity Assay

The cytotoxicity of all investigated compounds was assayed in all cells lines as previously described [12,13]. Briefly, confluent cells were treated with decreasing compound concentrations for 48h. The final DMSO concentration used was never greater than 0.5% *v*/*v*. Each experiment was performed in duplicate and repeated in two independent experiments. Cell viability was calculated using the CellTiter Glo 2.0 kit (Promega, Madison, WI, USA) and luminescence values signal obtained from cells treated with serial dilution of the compounds were measured through the GloMax^®^ Discover Multi mode Microplate Reader (Promega) and elaborated with GraphPad PRISM software version 9.0 (La Jolla, San Diego, CA, USA). The half-maximal cytotoxic concentration (CC_50_) was calculated using a non-linear regression analysis of the dose–response curves and the ECanything GraphPad function. A non-toxic dose of each compound was used as the starting concentration in the antiviral activity assay.

### 3.5. Biology—Viruses

#### 3.5.1. Viruses

The New Guinea C DENV serotype 2 and the WNV lineage 1 (Italy/2009) strains were kindly provided by the Istituto Superiore di Sanità (Rome, Italy), while the SARS-CoV-2 strain belonging to lineage B.1 (EPI_ISL_2472896) was kindly provided by the Department of Biomedical and Clinical Sciences Luigi Sacco, University of Milan (Italy). Once expanded in VERO E6 (African green monkey kidney cell line, ATCC catalog. n. CRL-1586), DENV, WNV and SARS-CoV-2 viral stocks were stored at −80 °C and titrated as previously described [12,13]. HIV-1 wild-type reference strain NL4-3 (catalog. n. ARP2006) was obtained through the NIH AIDS Reagent Program and the viral titer was calculated in TZM-bl cells through the detection of β-galactosidase expression.

#### 3.5.2. Antiviral Assays

##### Flaviviruses and SARS-CoV-2

To determine the antiviral activity of candidate compounds against DENV, WNV and SARS-CoV-2, a direct yield reduction assay based on the infection of cells in the presence of serial drug dilutions was performed as previously described with minor modifications [13]. Briefly, Huh7 or Caco-2 cells, pre-seeded in 96-well format, were infected with DENV and WNV viral stocks at 0.005 multiplicity of infection (MOI) or with SARS-CoV-2 at 0.004 MOI. After 1h of adsorption of the virus at 37 °C, viral inoculum was removed and serial dilutions of each tested compound, starting from the not-toxic dose, were added to the infected cells. After 48 h of incubation for DENV and WNV and 72 h for SARS-CoV-2, the antiviral activity was measured on the cell monolayer using immunodetection assay (IA), as previously described [13].

Absorbance was measured at 450 nm optical density (OD450) using the Absorbance Module of the GloMax^®^ Discover Multimode Microplate Reader (Promega). In each plate the suitable reference compound, a mock control (uninfected cells) and a virus control were included. Each IA run was validated when the OD450 values of virus control showed an OD450 > 1. All drug concentrations were tested in duplicate in two independent experiments. In each plate, SOF and REM were used as reference compounds against flaviviruses and SARS-CoV-2, respectively. Infected and uninfected cells without drugs were used to calculate the 100% and the 0% of viral replication, respectively. The half-maximal inhibitory concentration (IC_50_) was calculated through a non-linear regression analysis of the dose–response curves generated with GraphPad PRISM software version 9 (La Jolla, CA, USA). The Selectivity Index (SI) of the compounds was calculated as the ratio between the CC_50_ and the IC_50_.

##### HIV-1

The antiviral activity of investigated compounds was evaluated by measuring the IC_50_ values against the HIV-1 wild-type reference strain NL4-3 in a TZM-bl cell line-based phenotypic assay named BiCycle Assay [14]. The method includes a first round of infection in H9 cells at 0.08 MOI in the presence of the serial dilution of compounds in a 96-well plate. In each plate the reference compound, the mock control (uninfected cells) and the virus control were included. After 72 h, 50 µl of supernatants from each well were used to infect the TZM-bl cell line, which allows the quantitative analysis of HIV-1 infection by measuring the expression of the luciferase gene integrated in the genome of the cells under the control of the HIV-1 LTR promoter. After 48 h, dose–response curves were generated by measuring reporter gene expression in each well by using Bright-Glo Luciferase Assay (Promega) through the GloMax^®^ Discover Multimode Microplate Reader (Promega). Relative luminescence units measured in each well were elaborated with the GraphPad PRISM software version 9 to calculate IC_50_ values.

### 3.6. In Vitro ADME

#### 3.6.1. UV/LC-MS Method

For the quantitative analysis, a UV/LC-MS system was used. LC analysis was performed through an Agilent 1260 Infinity HPLC-DAD system (Agilent Technologies, Palo Alto, CA, USA), which constituted of a vacuum solvent degassing unit, a binary high-pressure gradient pump, and a UV detector, that was connected to an Agilent MSD 6130 system (Agilent Technologies, Palo Alto, CA, USA). The Agilent 1260 series mass spectra detection (MSD) single-quadrupole instrument was equipped with the orthogonal spray API-ES (Agilent Technologies, Palo Alto, CA, USA). Nitrogen was used as a nebulizing and drying gas. Chromatographic separation was performed using a Phenomenex Kinetex EVO C18-100 Å (150 × 4.6 mm, 5 μm particle size) at 25 °C and a gradient elution with a binary solution; eluent A was H_2_O, while eluent B consisted of ACN (both eluents were acidified with FA 0.1% *v*/*v*). The analysis started with 0% of B for 1 min, then rapidly increased up to 80% of B in the 15 min remaining until 19 min; finally, in one minute, it returned to the initial conditions of 100% of A. The analysis was performed at a flow rate of 0.6 mL/min. UV detection was monitored at 254 nm. Spectra were acquired over the scan range *m*/*z* 100–1500 in positive mode.

#### 3.6.2. Kinetic Aqueous Solubility

DMSO-stock compounds’ solutions were diluted with Mill-Q H_2_O to a final concentration of 200 μM. The percentage of DMSO never exceeded 2% *v*/*v*. Samples were incubated under gentle shaking at 25 °C (RT) conditions for 3 h. The suspensions were filtered using a 0.45 µm nylon filter (Acrodisc, VWR, Radnor, PE, USA), and the amount of solubilized compound was determined with the HPLC-UV-MS method reported above. The quantification of the solubilized compound was made with the appropriate calibration curve realized with stock solutions in DMSO (0.1–100 µg/mL). The limit of detection (LOD) was quantified at 0.1 µg/mL.

#### 3.6.3. Parallel Artificial Membrane Permeability Assay (PAMPA)

To evaluate the apparent permeability of tested compounds, a DMSO stock solution [1 mM] of each derivative was prepared and then diluted 1:1 *v*/*v* with phosphate buffer (PBS 25 mM, pH 7.4) in order to make the donor solutions. According to the protocol already published [9,15], the gastrointestinal (GI) phospholipidic bilayer was mimed through adding a 1% *w*/*v* dodecane solution of phosphatidylcholine (PC). The sandwich plates were assembled and incubated for 5 h at RT. At the end time point, the amount of compound passed through the phospholipid bilayer was measured using HPLC-UV/MS. Finally, apparent permeability (Papp) and membrane retention (MR%) were calculated as previously described [8].

#### 3.6.4. Metabolic Stability Assay

A DMSO solution of selected compounds was incubated in the presence of phosphate buffer (25 mM, pH 7.4), human liver microsomal protein (200 µg/mL), and an NADPH regenerating system in MgCl_2_ (48 mM) at a final concentration of 50 µM. The metabolic reactions were conducted for 1 h under shaking at 37 °C and then stopped by adding cold acetonitrile (ACN). Centrifuging the mixtures at 5000 rpm, the supernatant was separated, dried under nitrogen flow, and finally suspended in methanol (MeOH). The amount of the parent drug and the metabolites were determined as previously described [14].

#### 3.6.5. Plasma Stability Assay

A DMSO solution of each compound was incubated at a final concentration of 100 µM at 37 °C under shaking with HEPES buffer (25 mM, 140 mM NaCl, pH 7.4) and human plasma. At time 0 and after 24 h, samples were collected from the reaction mixtures and treated with cold ACN. After centrifugation at 5000 rpm for 10 min, the supernatant was collected, and the amount of unmodified compound was determined using the HLPC-UV/MS method described above. Modified compounds were calculated using time zero as 100% of the unmodified compound.

## 4. Conclusions

In conclusion, two small libraries of derivatives containing pyrimidine and pyridine privileged scaffolds were synthesized using a scaffold morphing approach, starting from previously obtained triazines. Newly synthesized compounds have been evaluated for their anticancer activity on lymphoma, hepatocarcinoma, and colon epithelial carcinoma cells. Three pyrimidine derivatives, **19a**, **19b**, and **21a,** were the most potent compounds synthesized demonstrating CC_50_ values in the low micromolar range against Huh7 and Caco-1 cell lines coupled with a good selectivity, especially in the case of **19b**. Two pyridine compounds, **33b** and **38b**, were the most selective derivatives synthesized.

Finally, the above-mentioned five azines were evaluated with in vitro ADME protocols showing a generally suboptimal aqueous solubility accompanied by good passive permeability and discrete percentages of membrane retention. From the metabolic point of view, neither in presence of liver microsomes nor with human plasma, tested compounds underwent massive degradation, thus allowing us to state that most of them manage to reach the potential biological target unaltered. Further studies aimed at the identification of a specific biological target for the newly synthesized azines are ongoing in our laboratories.

## Figures and Tables

**Table 1:** In vitro cytotoxicity and tumor selectivity index (TSI) of the synthesized compounds in a cell-based model ^1^.

		FB789 ^2^	H9 ^2^		Huh7 ^2^		Caco-2 ^2^	
Entry	CPD	CC_50_^3^	CC_50_	TSI	CC_50_ µM	TSI	CC_50_	TSI
**1**	**1**	30 ± 6	>100	<0.3	>100	<0.3	>400	<0.1
**2**	**2**	134 ± 11	>100	<1.3	>200	<0.7	>200	<0.7
**3**	**3**	250 ± 10	74 ± 6.2	3.4	45 ± 10	5.6	184 ± 11	1.4
**4**	**4**	11 ± 3	2 ± 1	5.5	9 ± 1	1.2	3 ± 1	3.7
**5**	**5**	27 ± 3	30 ± 5	0.9	45 ± 6	0.6	70 ± 6	0.4
**6**	**6**	100 ± 8	>100	<1.0	47 ± 5	2.1	22 ± 2	4.5
**7**	**7**	7 ± 2	2 ± 1	3.5	2 ± 0.4	3.5	0.8 ± 0.1	8.8
**8**	**8**	57 ± 7	>100	<0.6	>200	<0.3	>200	<0.3
**9**	**9**	50 ± 5	>100	<0.5	>200	<0.2	200	0.2

^1^ All experiments were conducted in duplicate in three independent experiments in FB789, H9, Huh7 and Caco-2 cells; ^2^ experiment read-out 48 h; ^3^ CC_50_, half-maximal compound cytotoxic concentration, expressed in micromolar (µM) range, as determined using Cell-Titer Glo kit (Promega, Madison, WI, USA). TSI, tumor selectivity index (ratio between CC_50_ on FB789 and CC_50_ on cancer cells H9, Huh7 and Caco-2). CPD = compound.

**Table 2:** Cytotoxicity (µM) and antiproliferative activity (TSI) of highly functionalized pyrimidine and pyridine compounds against H9, Huh7 and Caco-2 cell lines ^1^.

			FB789 ^2^	H9 ^2^		Huh7 ^2^		Caco-2 ^2^	
Entry	CPD	Ref. T. ^3^	CC_50_ ^4^	CC_50_ µM	TSI	CC_50_	TSI	CC_50_	TSI
**1**	**14a**	**1**	30 ± 6	>100	<0.3	82 ± 6	0.4	84 ± 7	0.4
**2**	**14b**	**3**	9 ± 1	14 ± 7	0.6	8 ± 4	1.1	11 ± 2	0.8
**3**	**19a**	**7**	14 ± 3	31 ± 8	0.5	1 ± 0.3	14.0	2 ± 1	7.0
**4**	**19b**	**9**	61 ± 9	48 ± 3.5	1.3	6 ± 1	10.2	4 ± 1	15.3
**5**	**21a**	**4**	10 ± 5	11 ± 2	0.9	3 ± 1	3.3	2 ± 0.4	5.0
**6**	**21b**	**6**	95 ± 1	>100	<1.0	67 ± 23	1.4	113 ± 4	0.8
**7**	**27a**	**1**	11 ± 3	76 ± 4	0.1	44 ± 5	0.3	29 ± 2	0.4
**8**	**27b**	**3**	81 ± 2	50 ± 4	1.6	39 ± 3	2.1	30 ± 2	2.7
**9**	**33a**	**7**	0.3 ± 0.3	43 ± 9	0.01	18 ± 5	0.02	12 ± 4	0.03
**10**	**33b**	**9**	390 ± 30	23 ± 7	17.0	21 ± 12	18.6	11 ± 9	35.5
**11**	**38a**	**4**	3 ± 1	37 ± 2	0.1	24 ± 14	0.1	19 ± 0.1	0.2
**12**	**38b**	**6**	90 ± 9	18 ± 4	5.0	9 ± 6	10.0	8 ± 3	11.3

^1^ Values are the means ± SD of experiments run in triplicate; ^2^ experiments read out at 48 h; ^3^ Ref. T. = reference triazine, triazine derivative from which the newly synthesized pyrimidine or pyridine are derived; ^4^ CC_50_, half-maximal compound cytotoxic concentration, expressed in micromolar (µM) units. TSI, tumor selectivity index (ratio between CC_50_ on FB789 and CC_50_ on cancer cells H9, Huh7 and Caco-2). CPD = compound.

**Table 3:** Kinetic water solubility of tested compounds.

CPD	µg/mL	LogS ^1^	Predicted LogS ^2^
**19a**	<0.1	-	−7.50
**19b**	<0.1	-	−7.82
**21a**	<0.1	-	−6.69
**33b**	<0.1	-	−7.87
**38b**	0.54	−5.87	−5.92

^1^ Log of Solubility reported as mol/L. ^2^ LogS data predicted with SwissADME.

**Table 4:** In vitro PAMPA permeability studies of tested compounds.

CPD	P_app_ ^1^	MR ^2^ (%)
**19a**	3.59 ± 0.21	20.91 ± 1.75
**19b**	5.64 ± 0.59	27.42 ± 1.22
**21a**	4.80 ± 0.73	26.03 ± 6.17
**33b**	1.38 ± 0.60	46.84 ± 4.99
**38b**	6.35 ± 0.67	20.99 ± 1.69

^1^ Apparent Permeability (P_app_) reported in cm/s × 10^−6^. ^2^ Membrane retention %.

**Table 5:** In vitro metabolic stability studies of tested compounds in the presence of liver microsomes and plasma.

CPD	Metabolic Stability (%)	Metabolite Formation (%) ^1^	Plasmatic Stability (%) ^2^
**19a**	>99.9	-	86.64 ± 0.17
**19b**	98.31 ± 0.11	M_1_ = 1.68 ± 0.11	>99.9
**21a**	>99.9	-	96.93 ± 2.73
**33b**	>99.9	-	81.01 ± 3.28
**38b**	97.93 ± 0.46	M_1_ = 2.06 ± 0.46	87.13 ± 0.48

^1^ M_1_ = M + OH (+16). ^2^ Plasma stabilities after 24 h of incubation were calculated as percentages of Time 0 taken as 100%.

## Data Availability

Data are contained within the article and Supplementary Materials.

## Supplementary Materials

The following supporting information can be downloaded at: https://www.mdpi.com/article/10.3390/molecules29071452/s1, Supplement S1: Nuclear Overhauser Effect Spectroscopy (NOESY) of pyrimidine intermediates **11** and **12**; Supplement S2: ^1^H NMR of pyridine intermediates **23** and **24**; Supplement S3: NMR spectra for highly decorated pyrimidines and pyridine derivatives.
