# Supplementary material for: Serendipitous Identification of Azine Anticancer Agents Using a Privileged Scaffold Morphing Strategy
Source: Molecules. 2024 Mar 24;29(7):1452. doi: 10.3390/molecules29071452 (PMC11013010; doi:10.3390/molecules29071452)

Supplementary material

Serendipitous Identification of Azine Anticancer Agents Using a Privileged Scaffold Morphing Strategy

Silvia Cesarini [1, †], Ilaria Vicenti [2,†], Federica Poggialini [3,†], Silvia Filippi [1,†], Eleonora Mancin [1], Lia Fiaschi [2], Elisa De Marchi [1], Federica Giammarino [2], Chiara Vagaggini [3], Bruno Mattia Bizzarri, Raffaele Saladino [1], Elena Dreassi [3], Maurizio Zazzi [2], Lorenzo Botta [1,*]

[1] Department of Biological and Ecological Sciences, University of Viterbo, Via S.C. De Lellis s.n.c., 01100 Viterbo, Italy; c.cesarinisilvia@gmail.com (S.C.); silvia.filippi@unitus.it (S.F.); eleonora.mancin98@gmail.com (E.M.); elisa.demarchi@unitus.it (E.D.M.); bm.bizzarri@unitus.it (B.M.B.); saladino@unitus.it (R.S.)

[2] Department of Medical Biotechnologies, University of Siena, 53100 Siena, Italy; ; ilariavicenti@gmail.com (I.V.); lia.fiaschi@unisi.it (L.F.); federica.giammari@gmail.com (F.G.); maurizio.zazzi@gmail.com (M.Z.)

[3] Department of Biotechnology, Chemistry, and Pharmacy (DBCF), University of Siena, 53100 Siena, Italy; poggialini5@student.unisi.it (F.P.); chiara.vagaggini@student.unisi.it (C.V.); elena.dreassi@unisi.it (E.D.)

* Correspondence: lorenzo.botta@unitus.it;

† These authors contributed equally to this work.

S1: Nuclear Overhauser Effect Spectroscopy (NOESY) of pyrimidine intermediates 11 and 12.

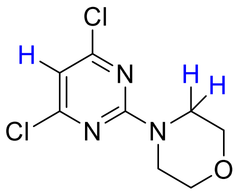

11

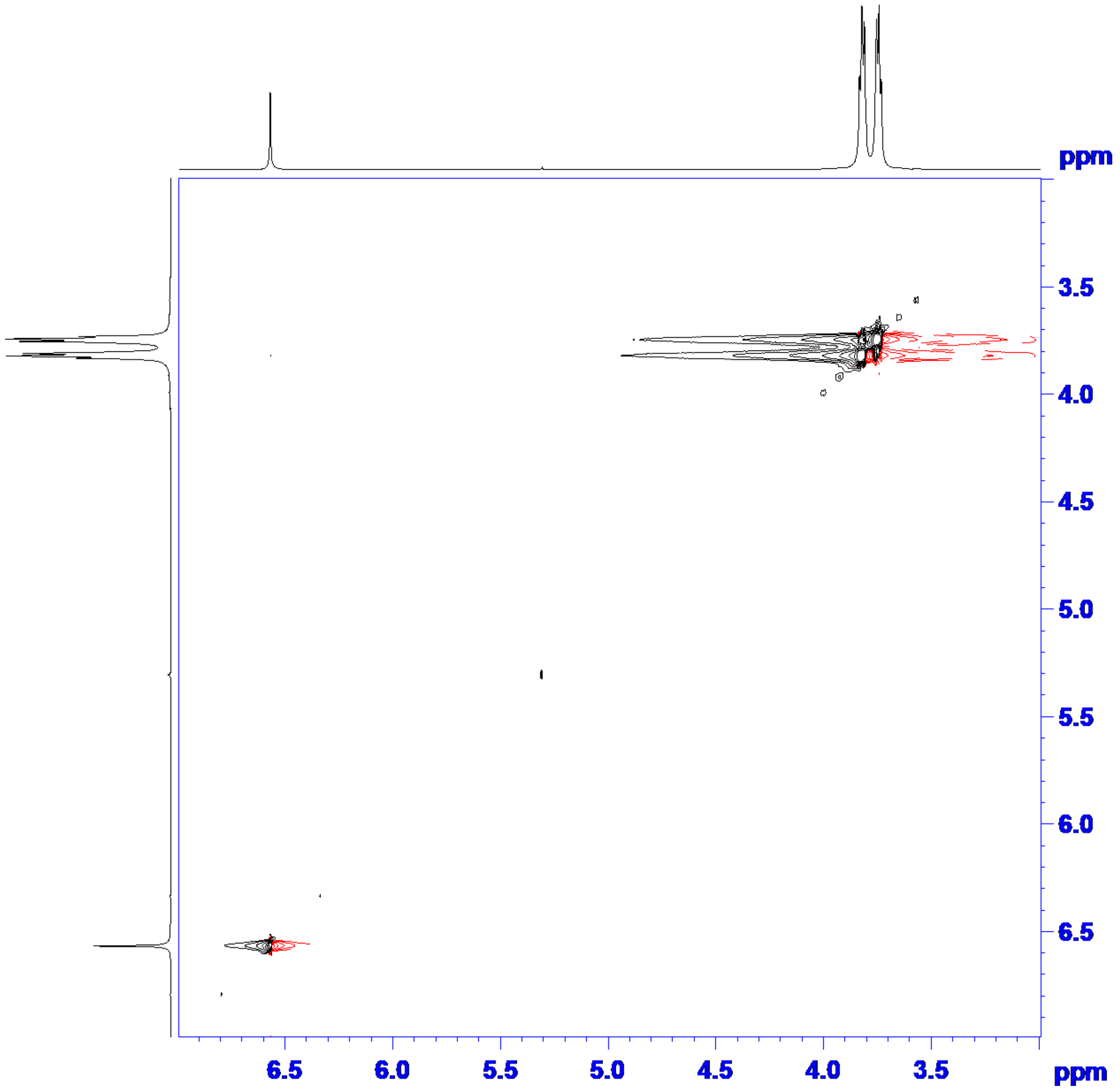

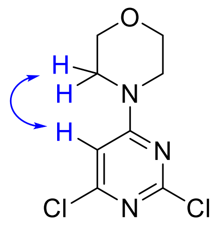

12

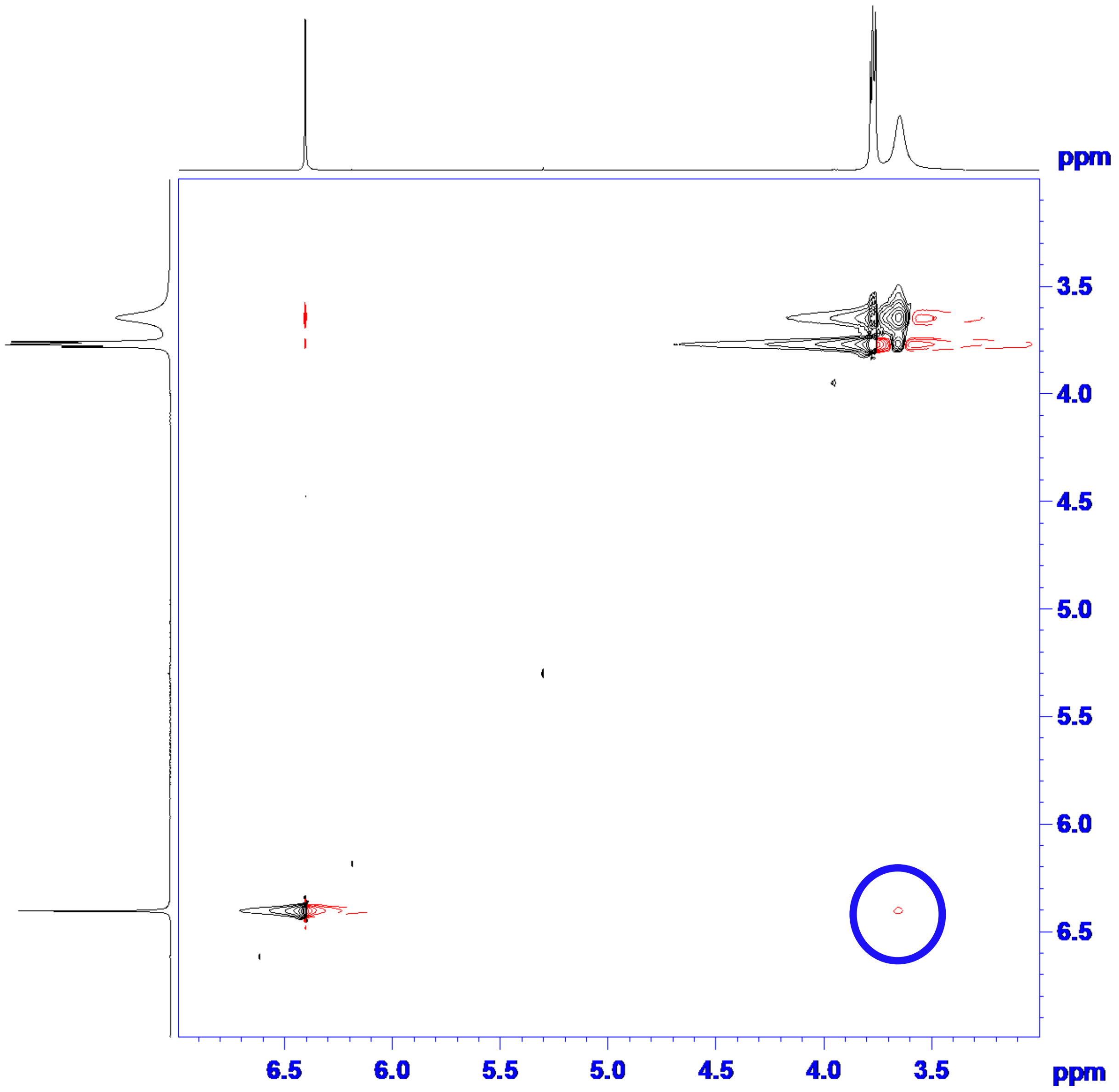

S2: ¹H NMR of pyridine intermediates 23 and 24.

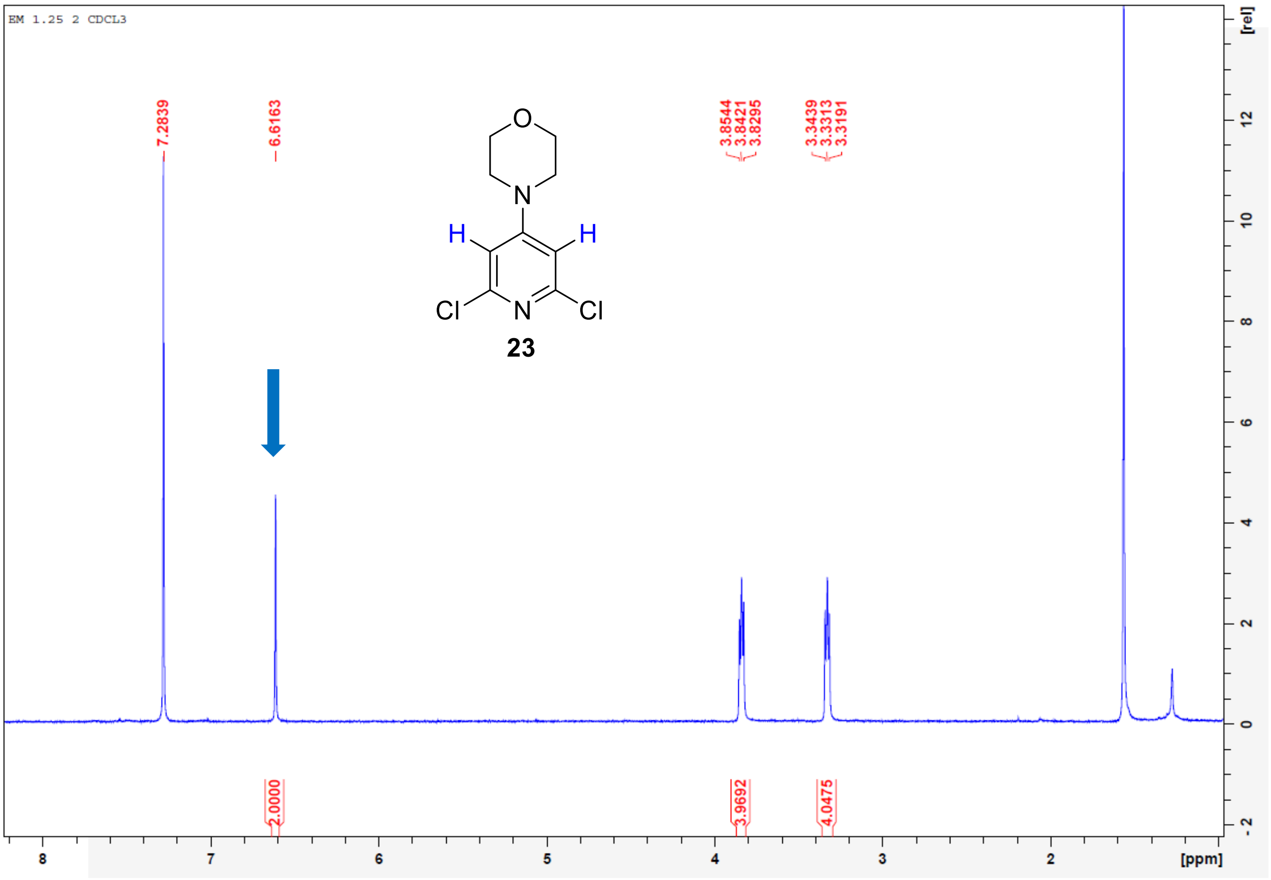

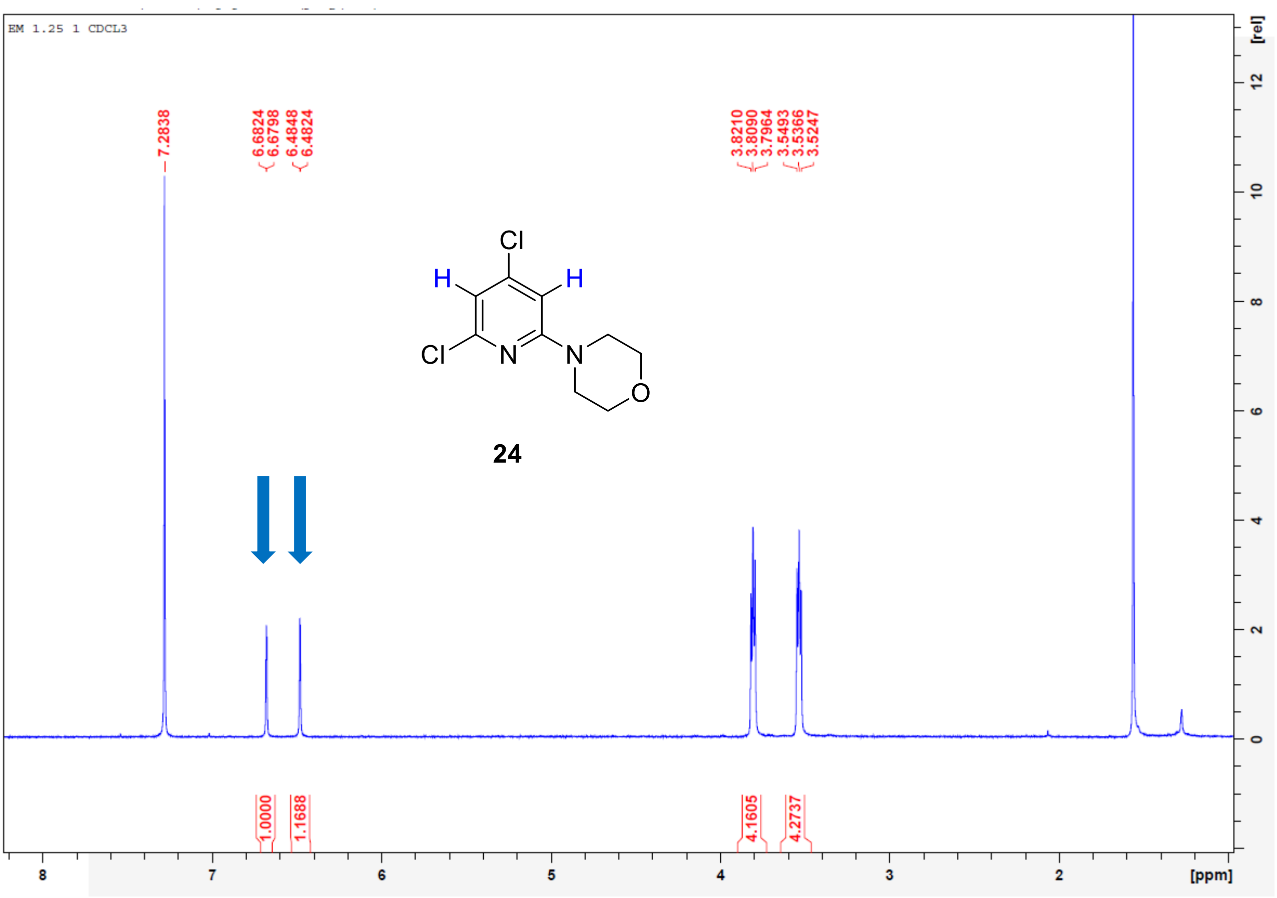
EM 1.25 1 CDCL3
7.2838
6.6824
6.6798
6.4848
6.4824
3.8210
3.8090
3.7964
3.5493
3.5366
3.5247
Cl
H
H
Cl
N
N
O
24
1.0000
1.1688
4.1605
4.2737
[rel]
12
10
8
6
4
2
0
8
7
6
5
4
3
2
[ppm]

S3: NMR spectra for highly decorated pyrimidines and pyridine derivatives
Pyrimidine 14a

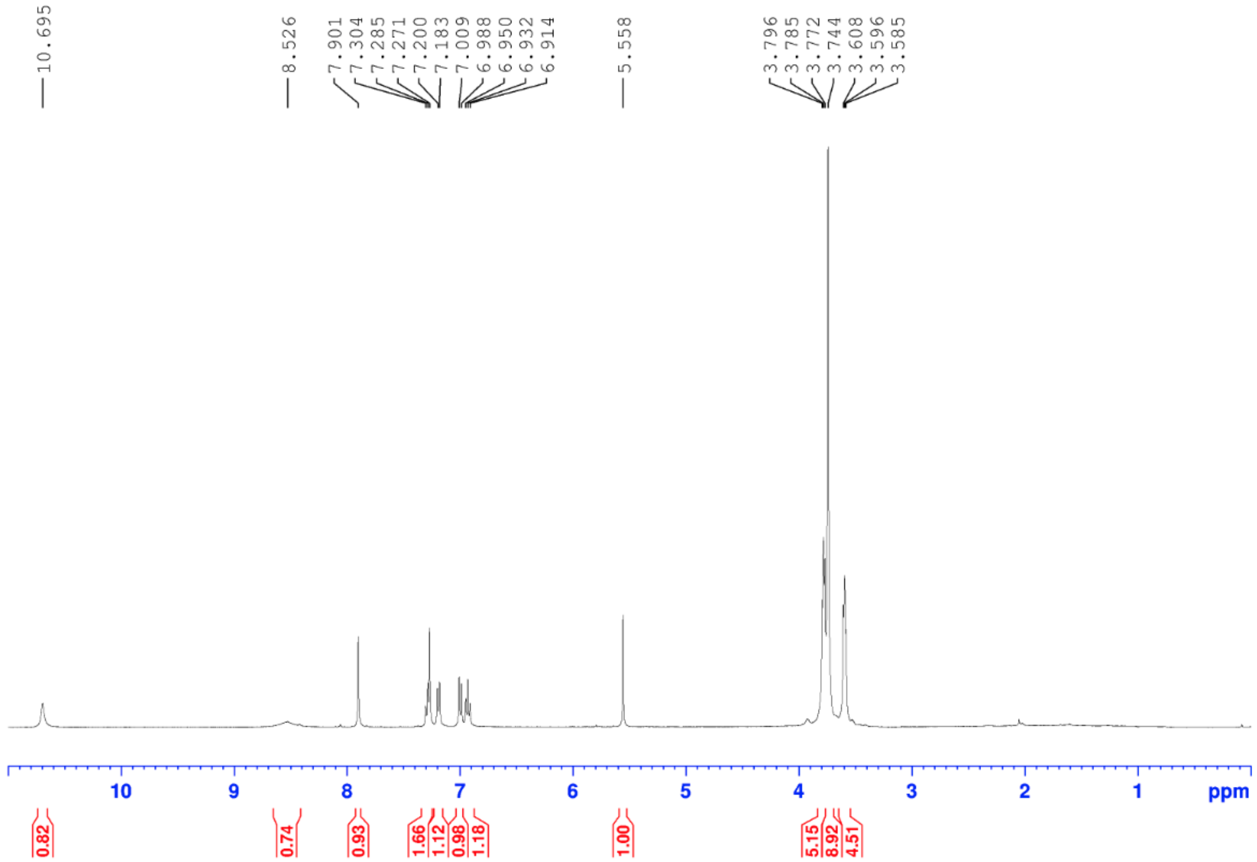

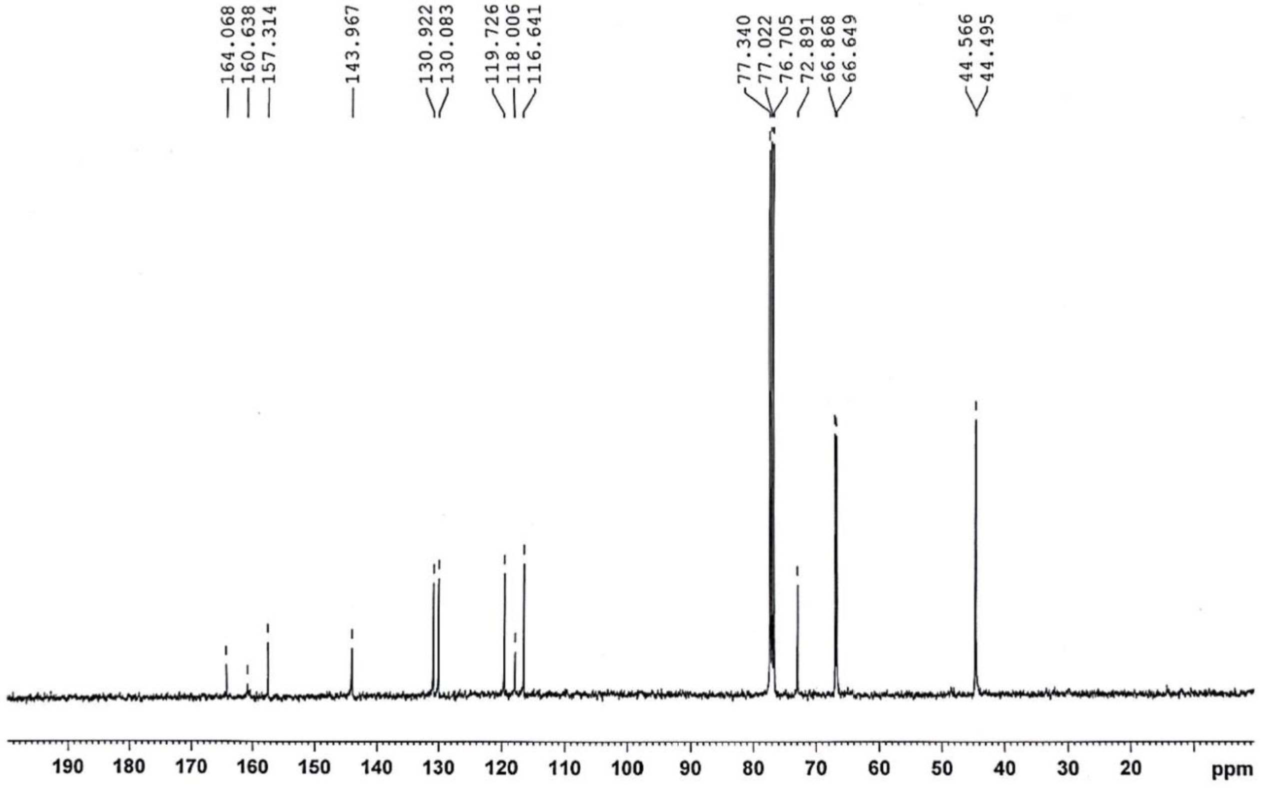

Pyrimidine 14b

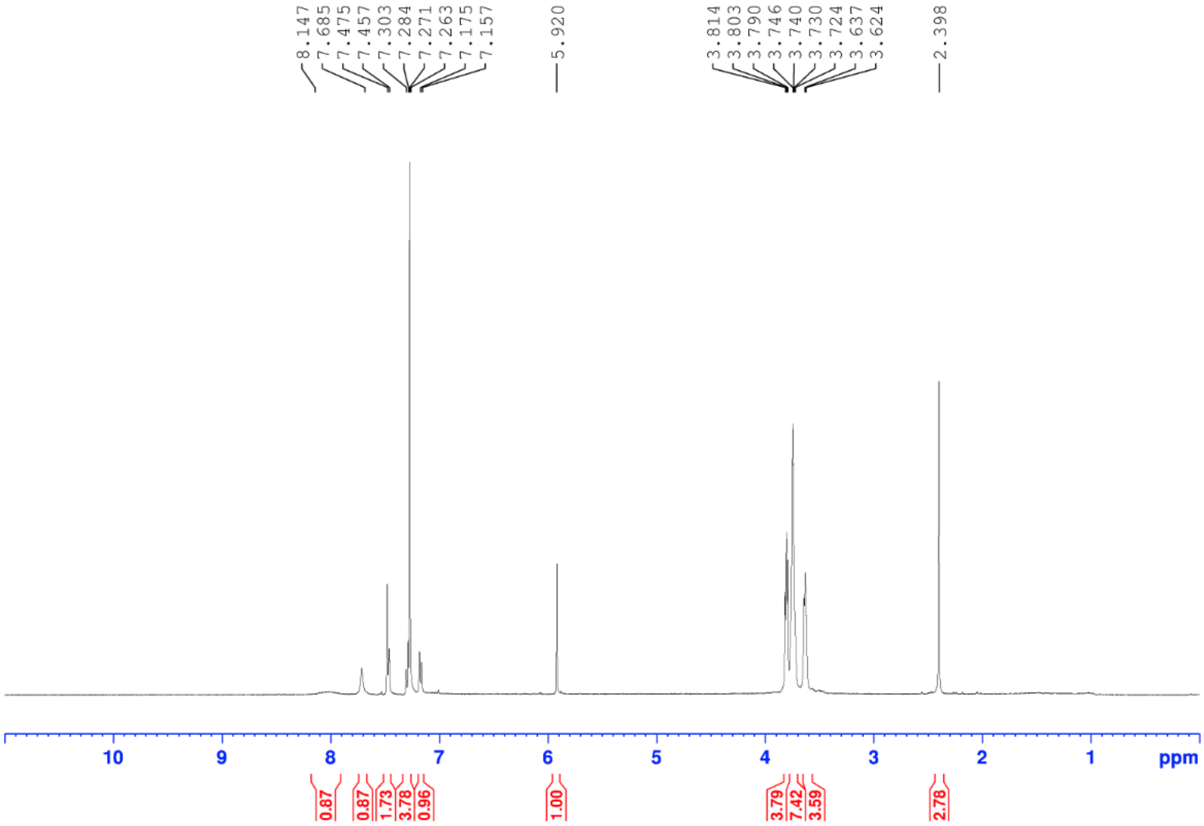

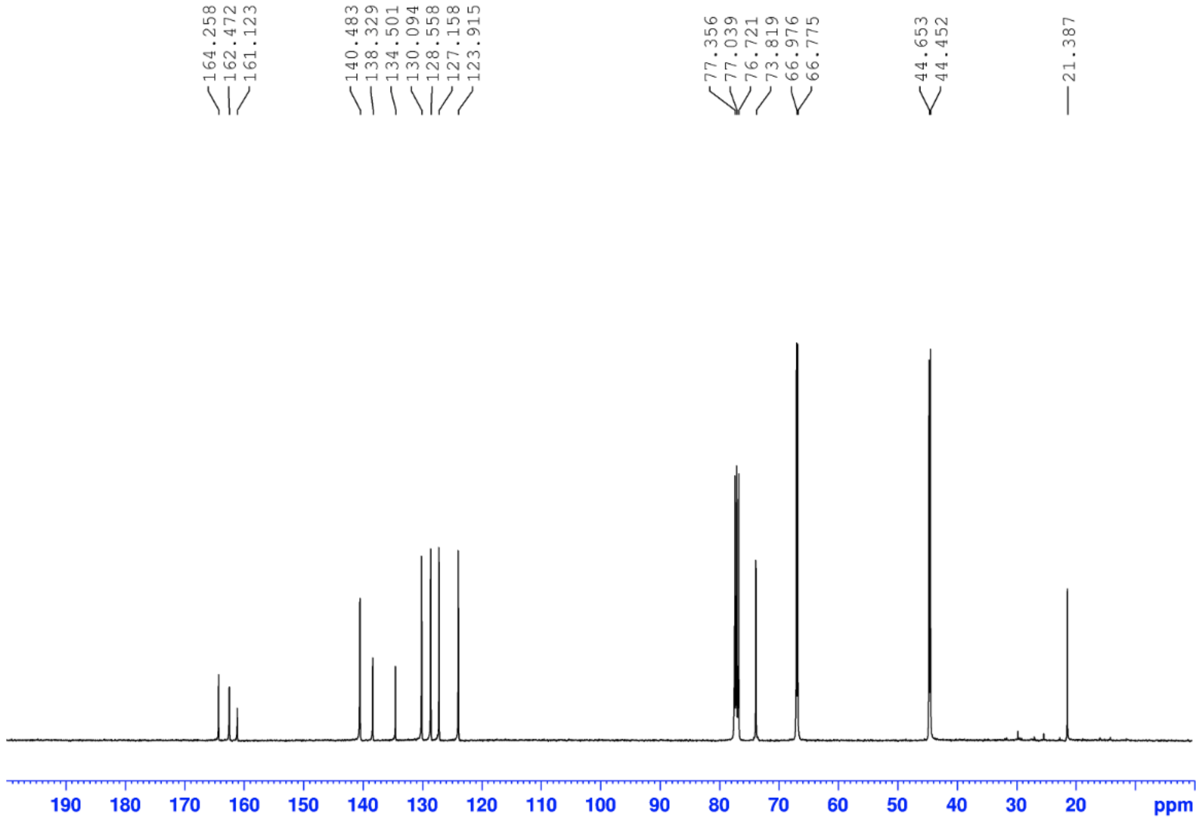

Pyrimidine 19a

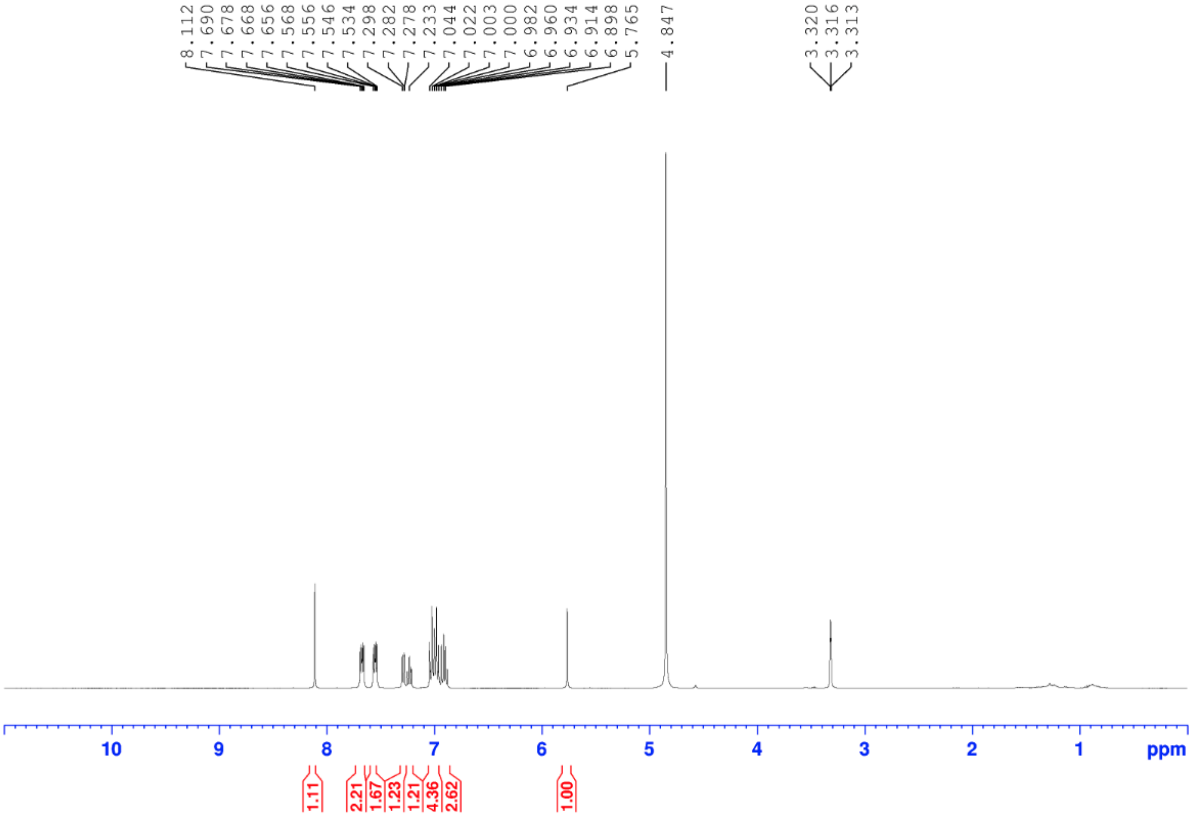

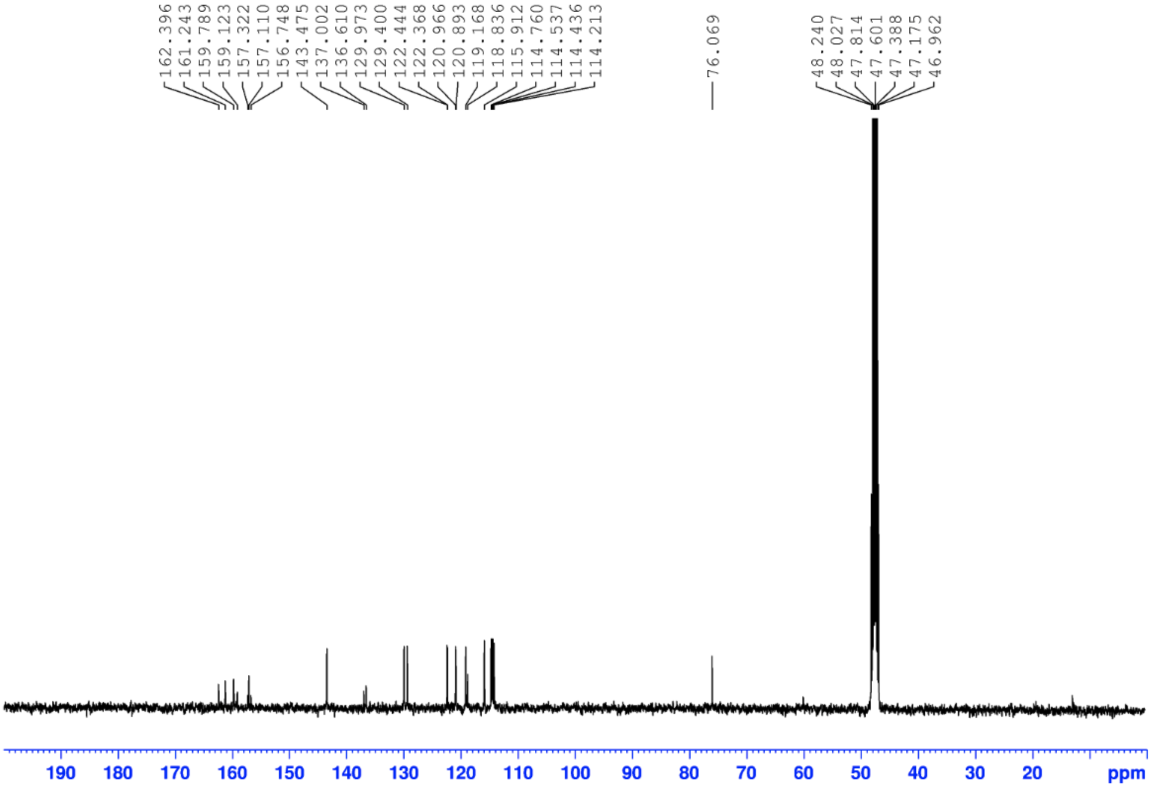

Pyrimidine 19b

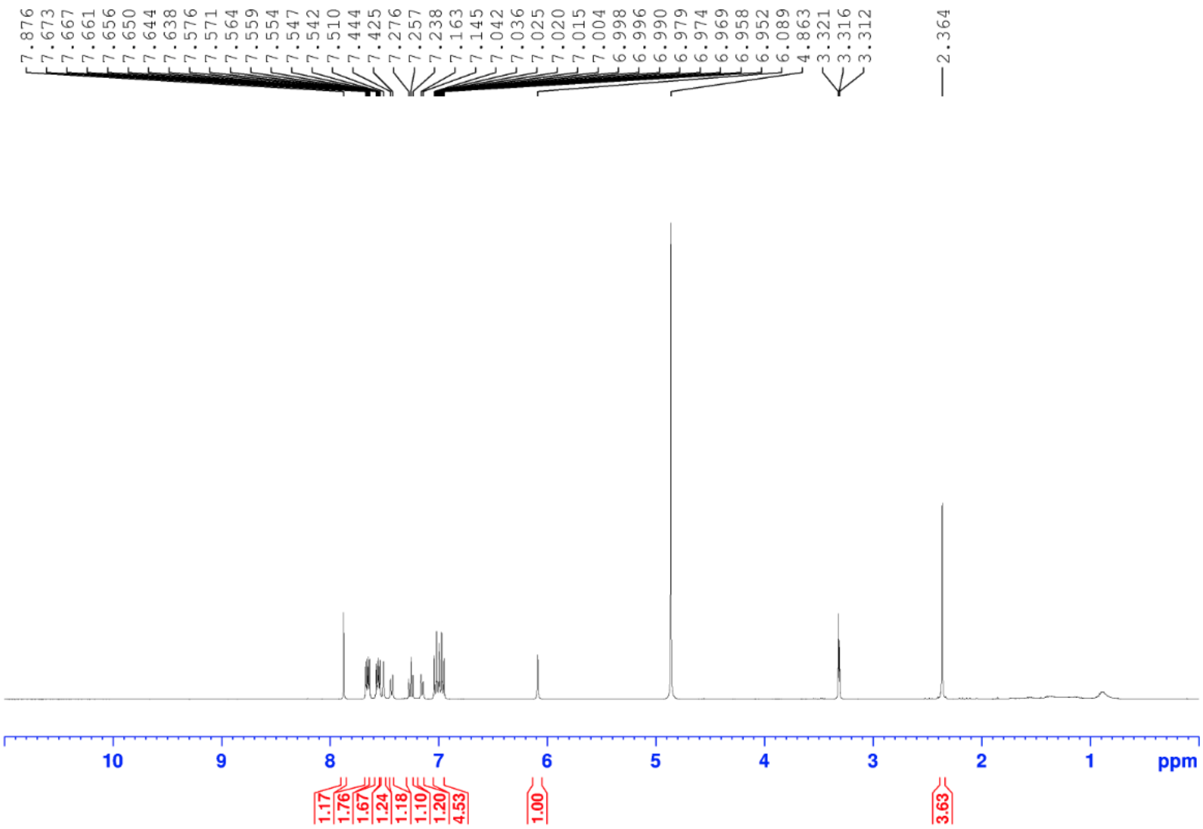

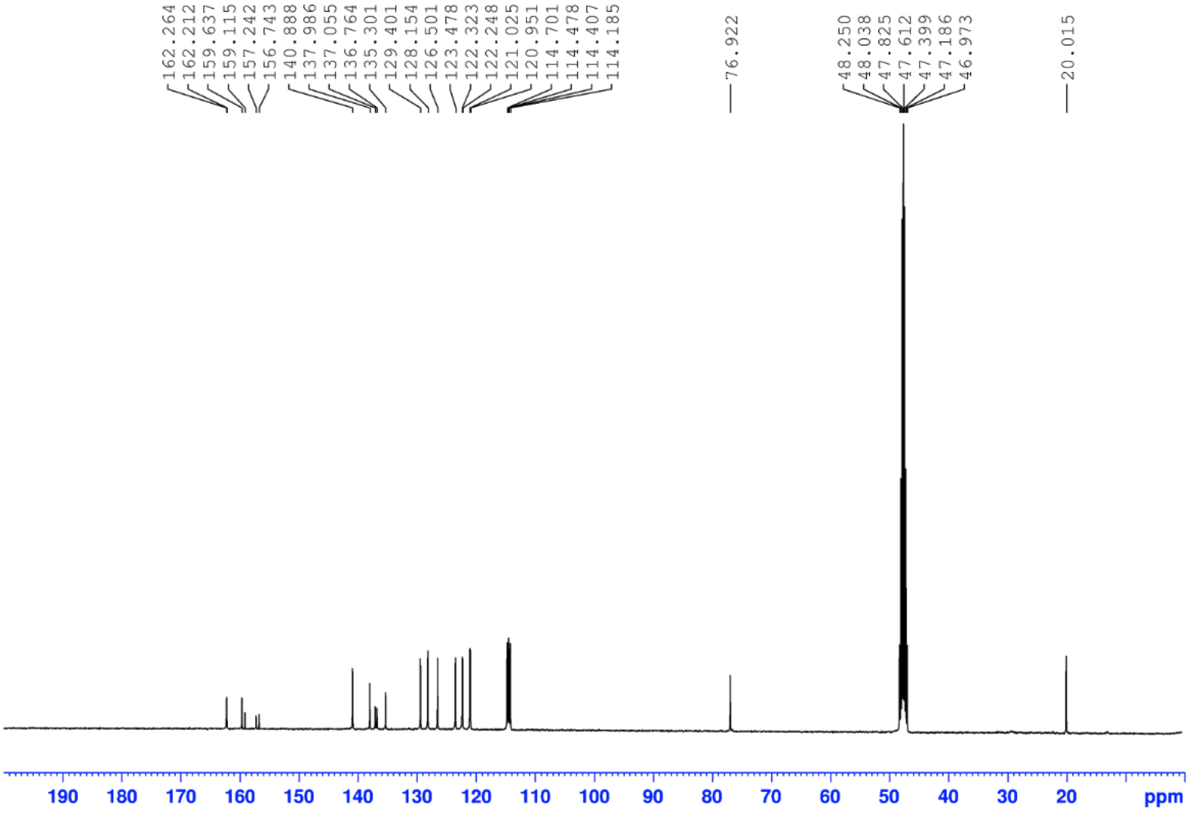

Pyrimidine 21a

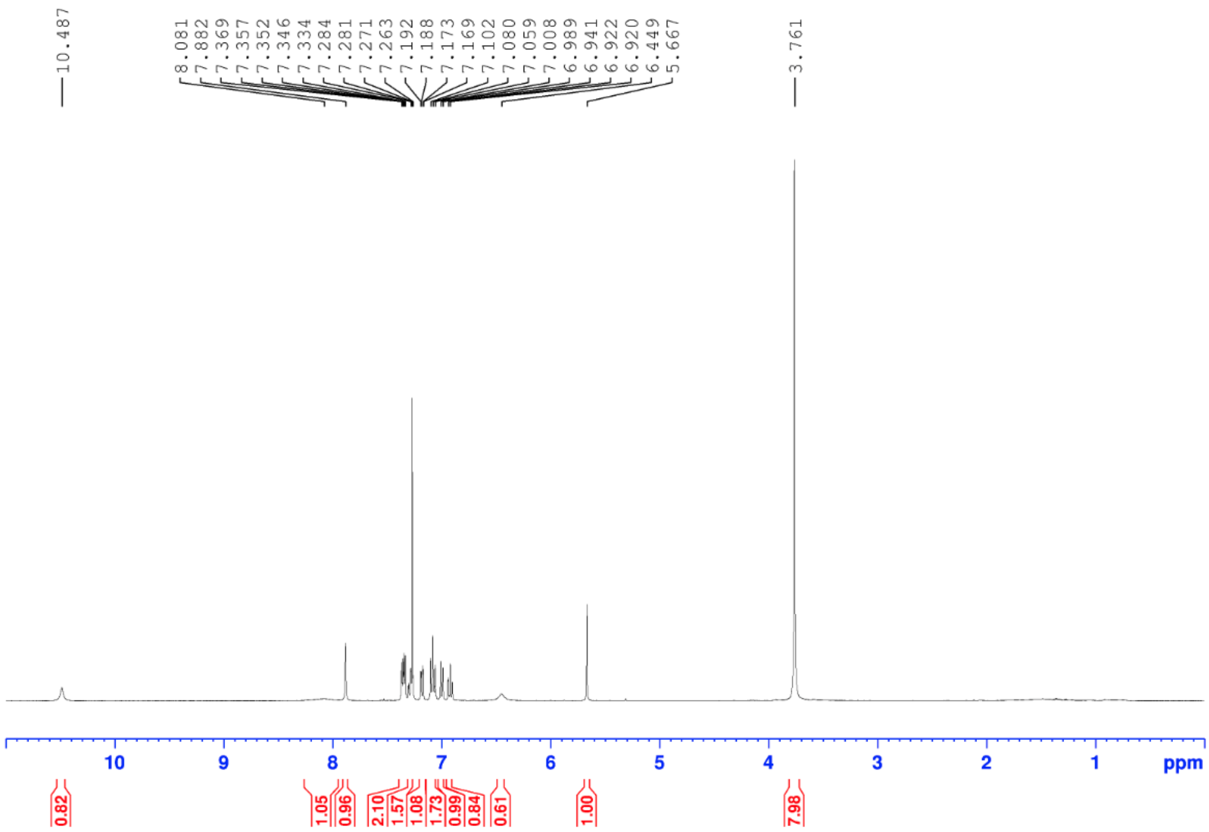

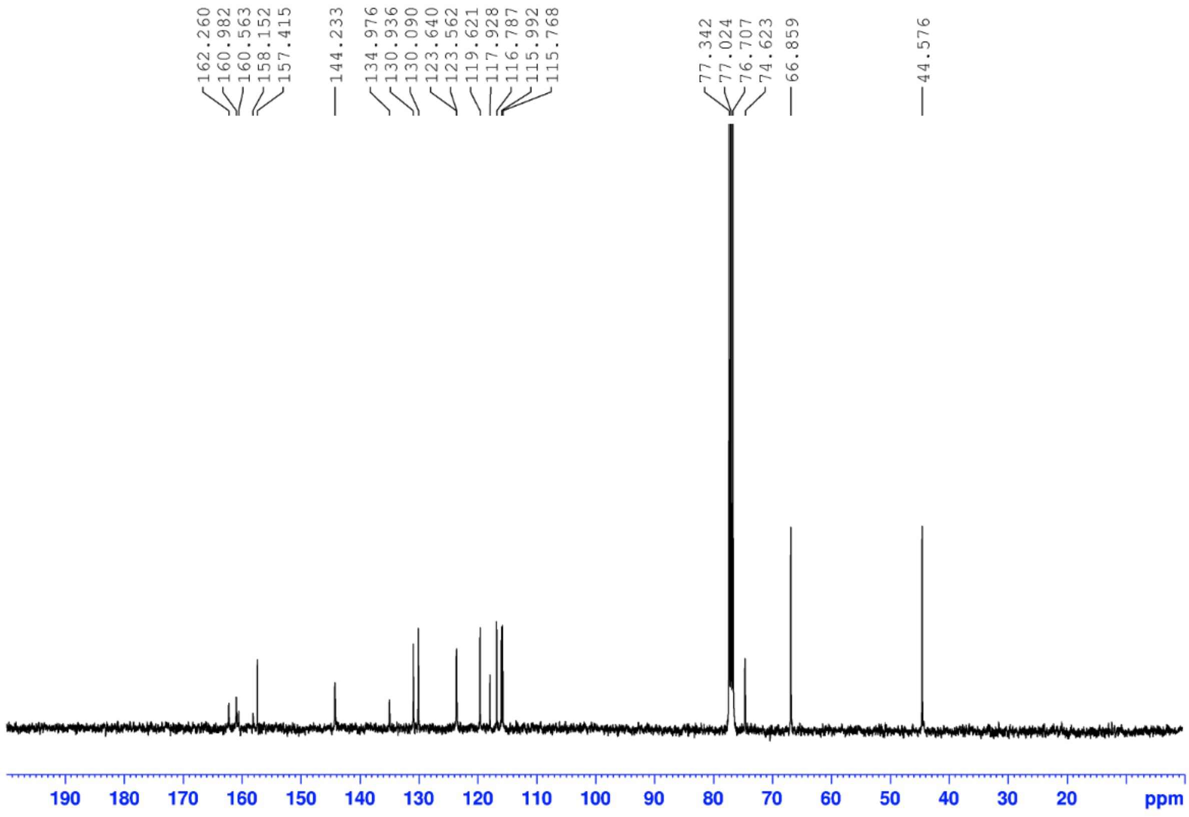

Pyrimidine 21b

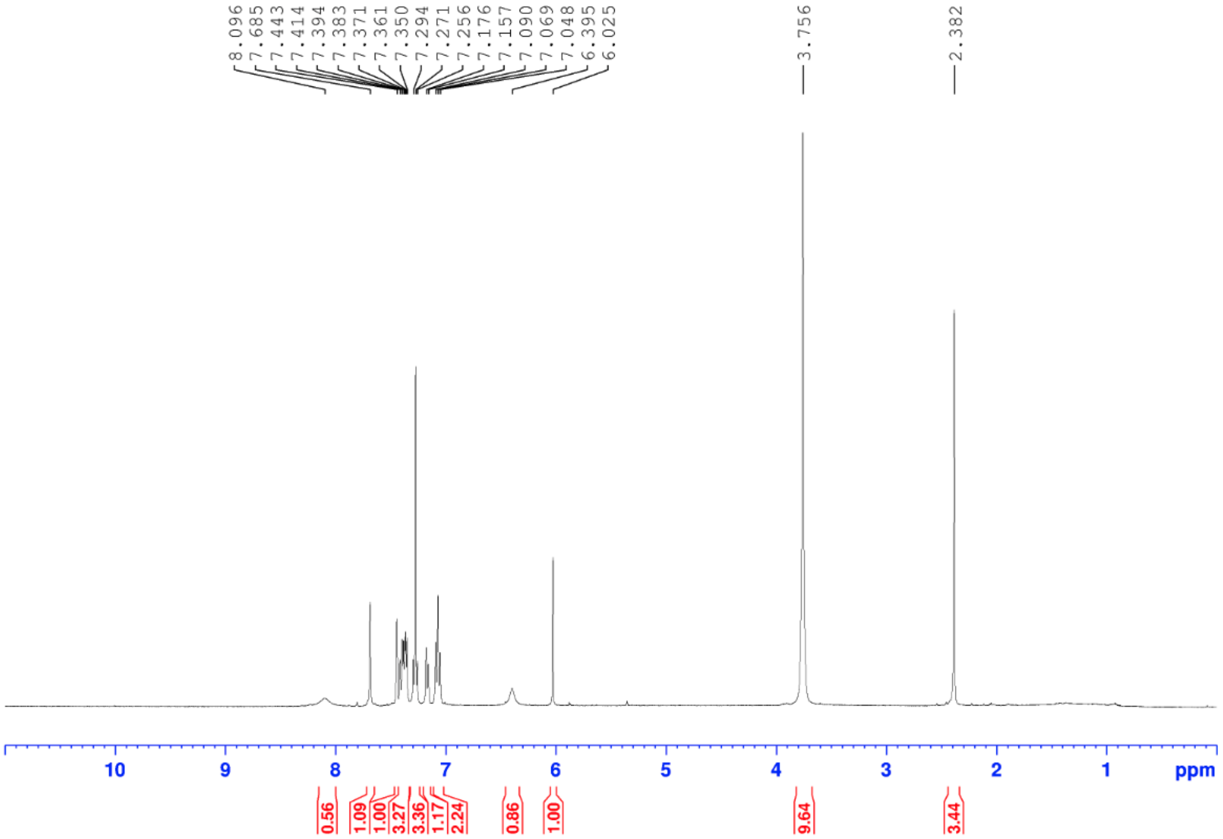

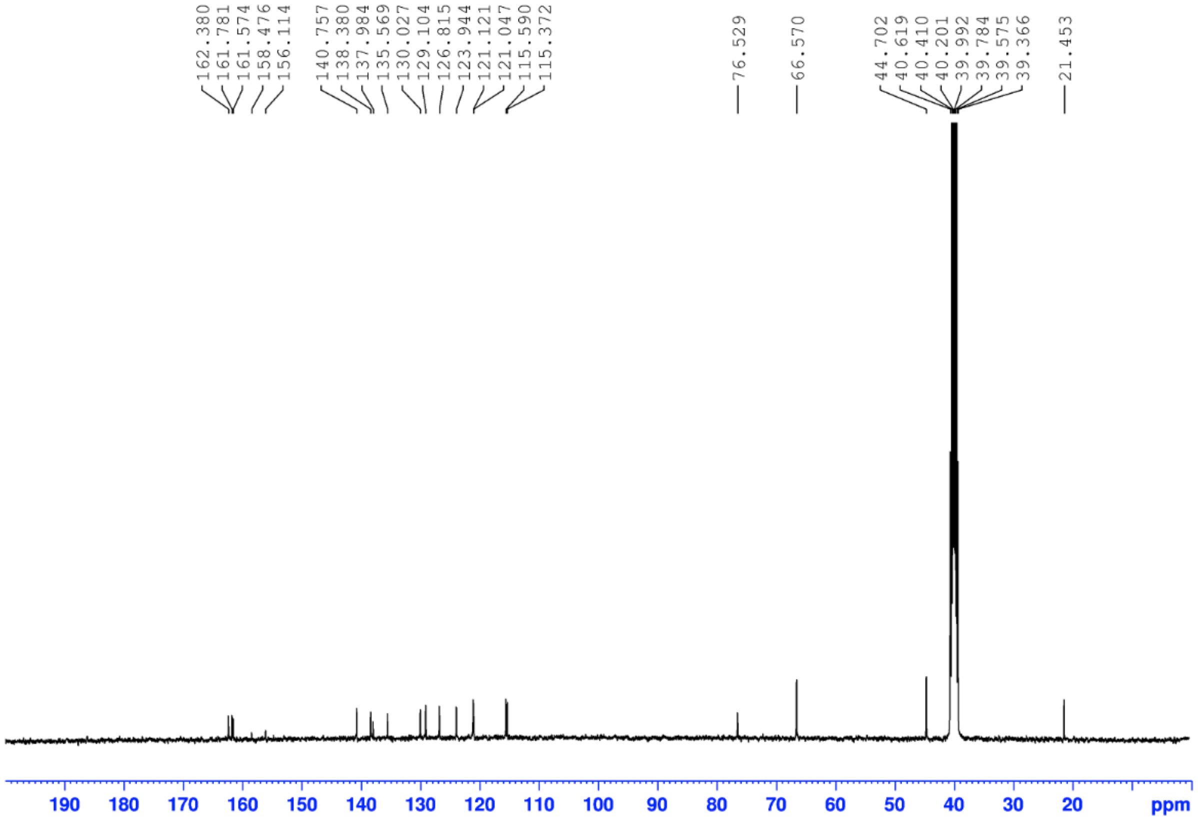

Pyridine 27a

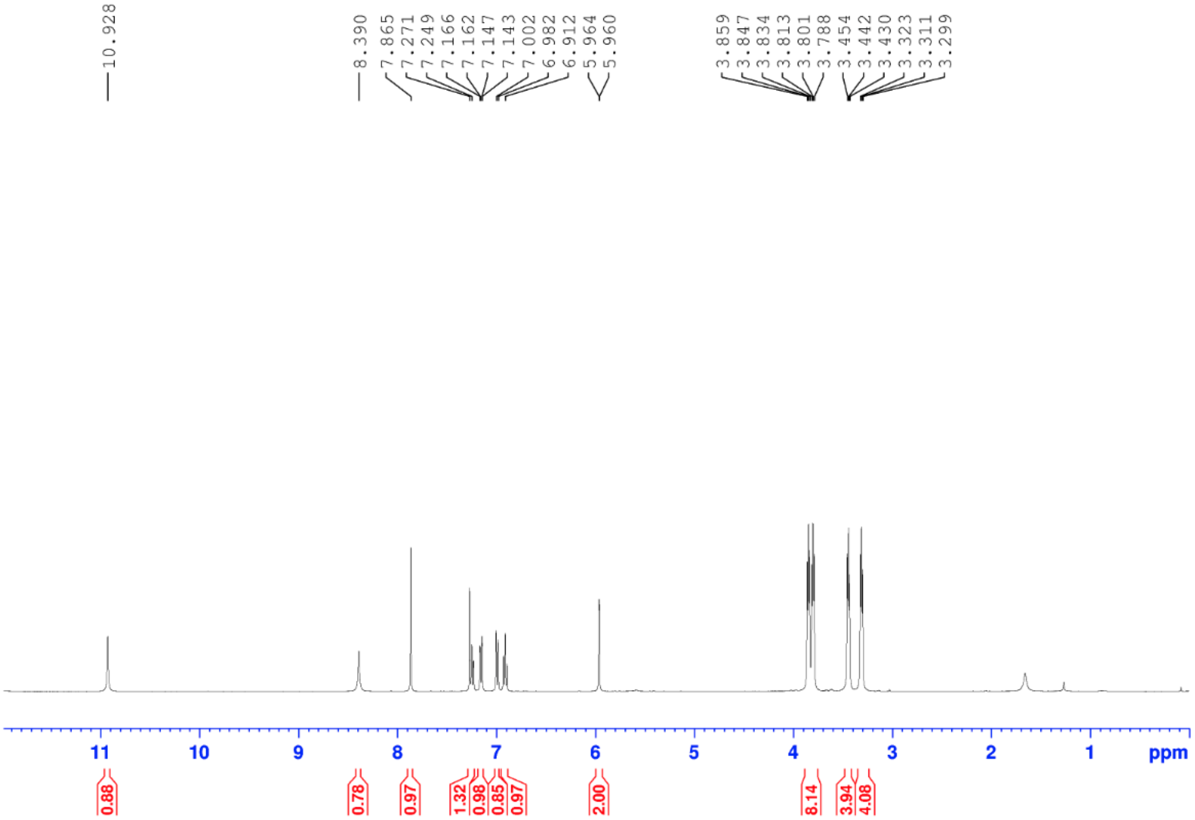

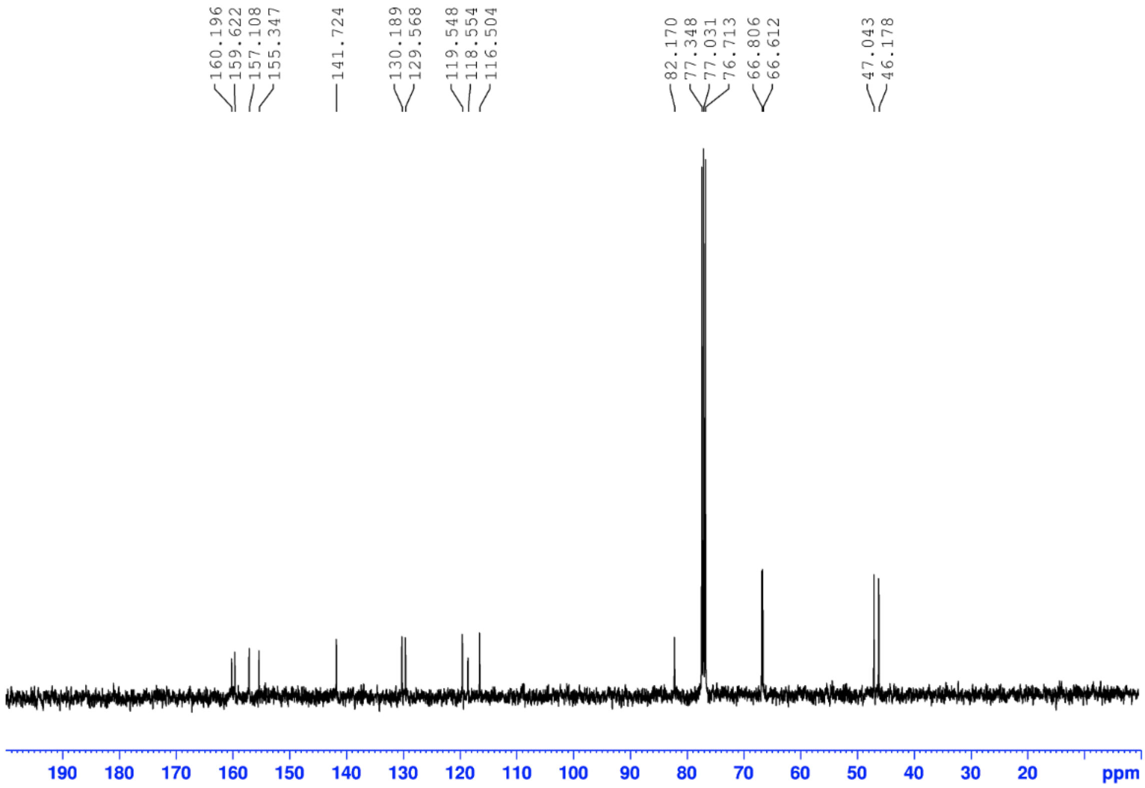

Pyridine 27b

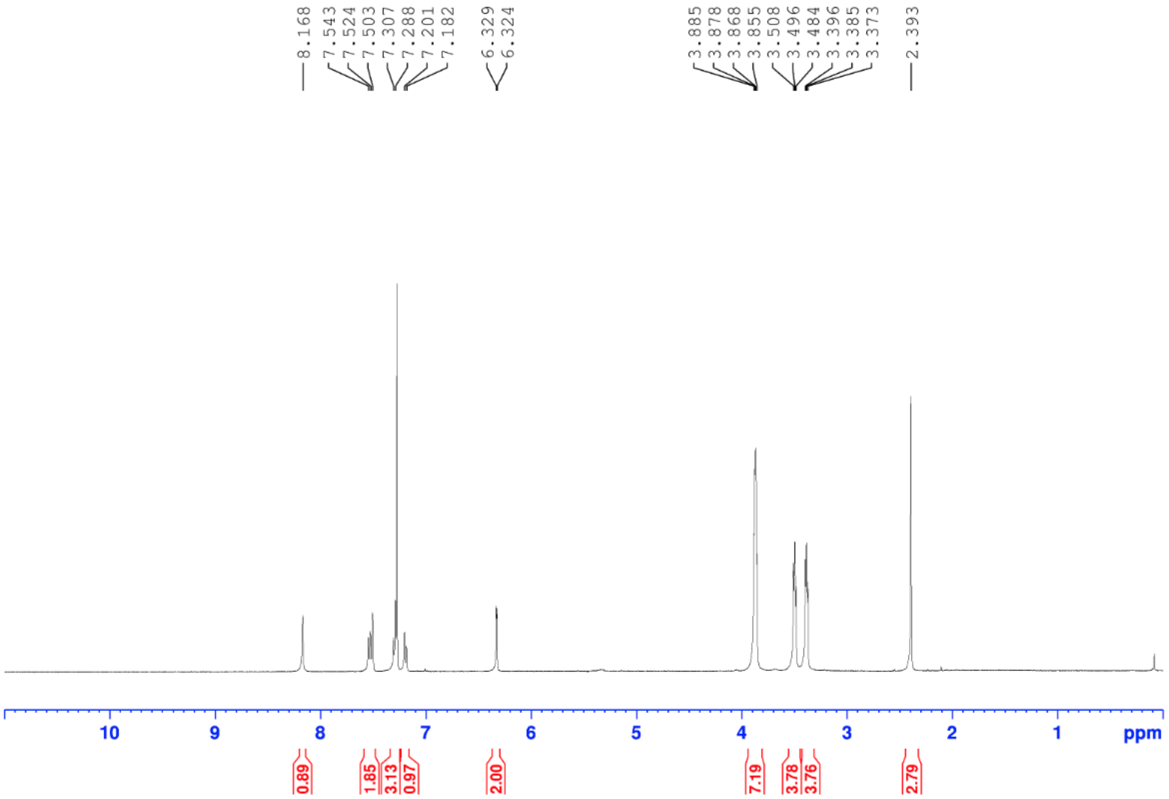

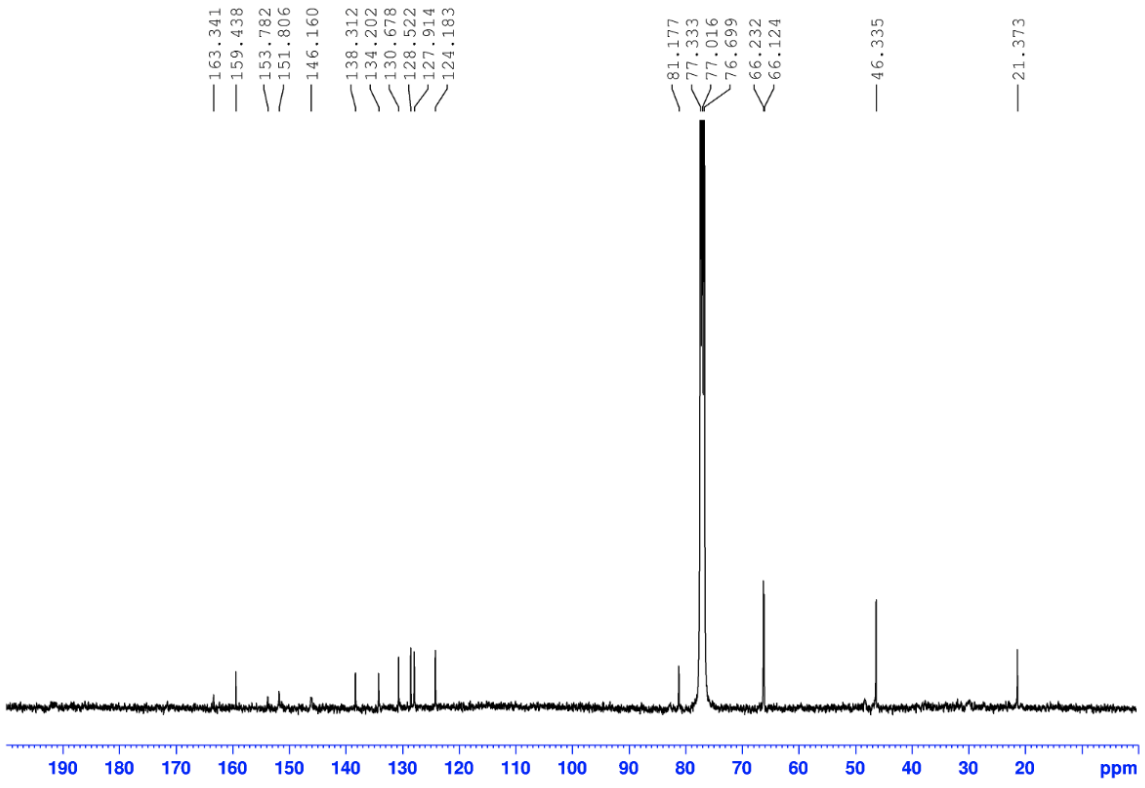

Pyridine 33a

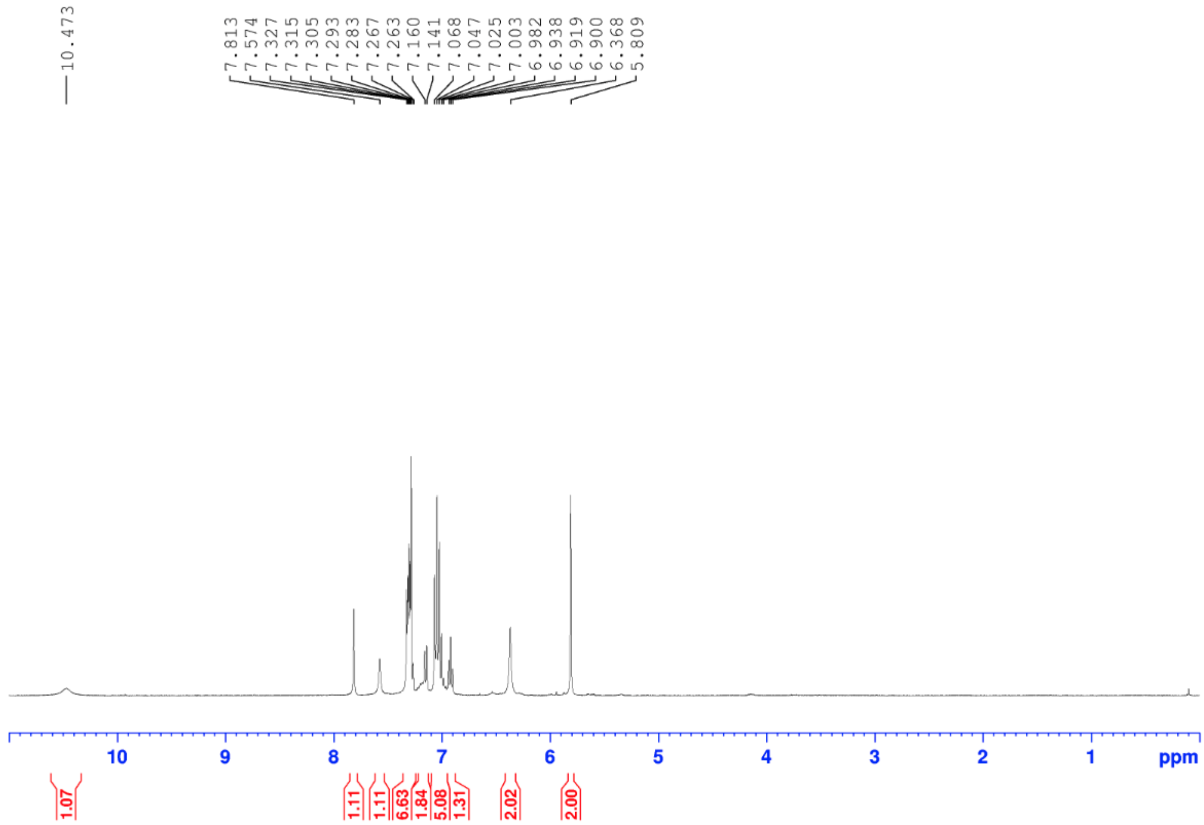

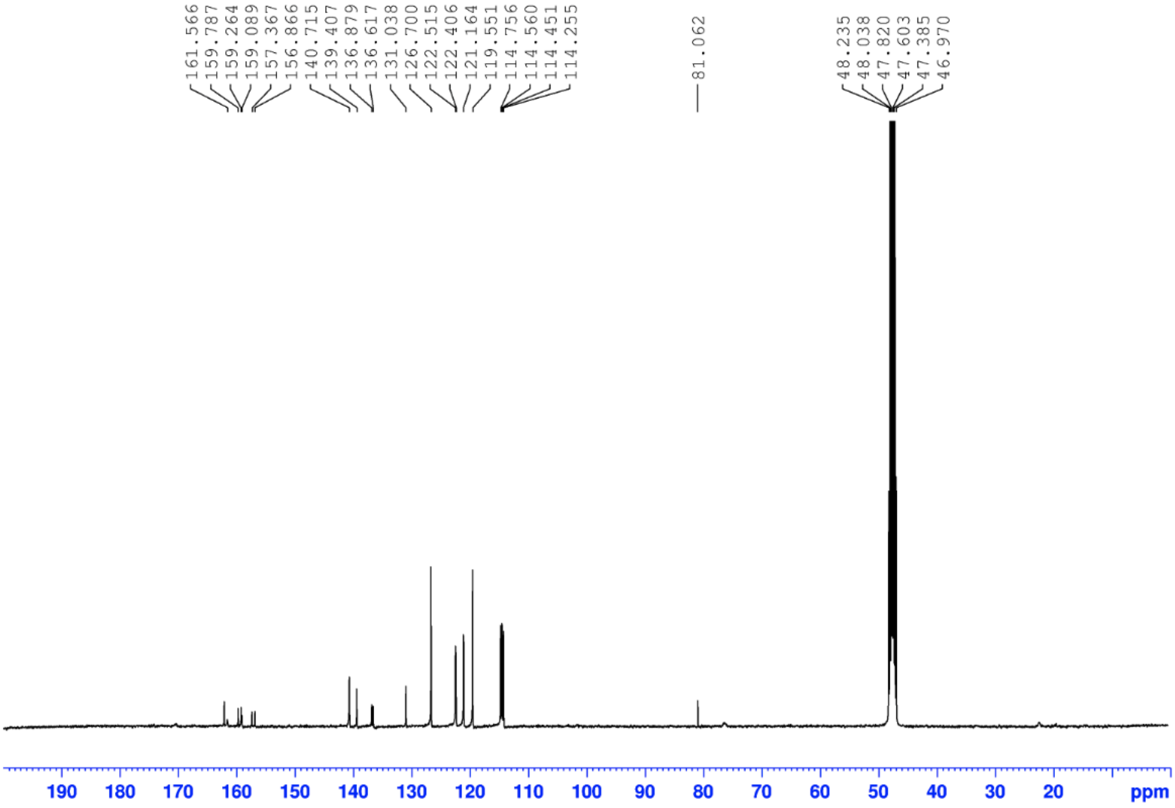

Pyridine 33b

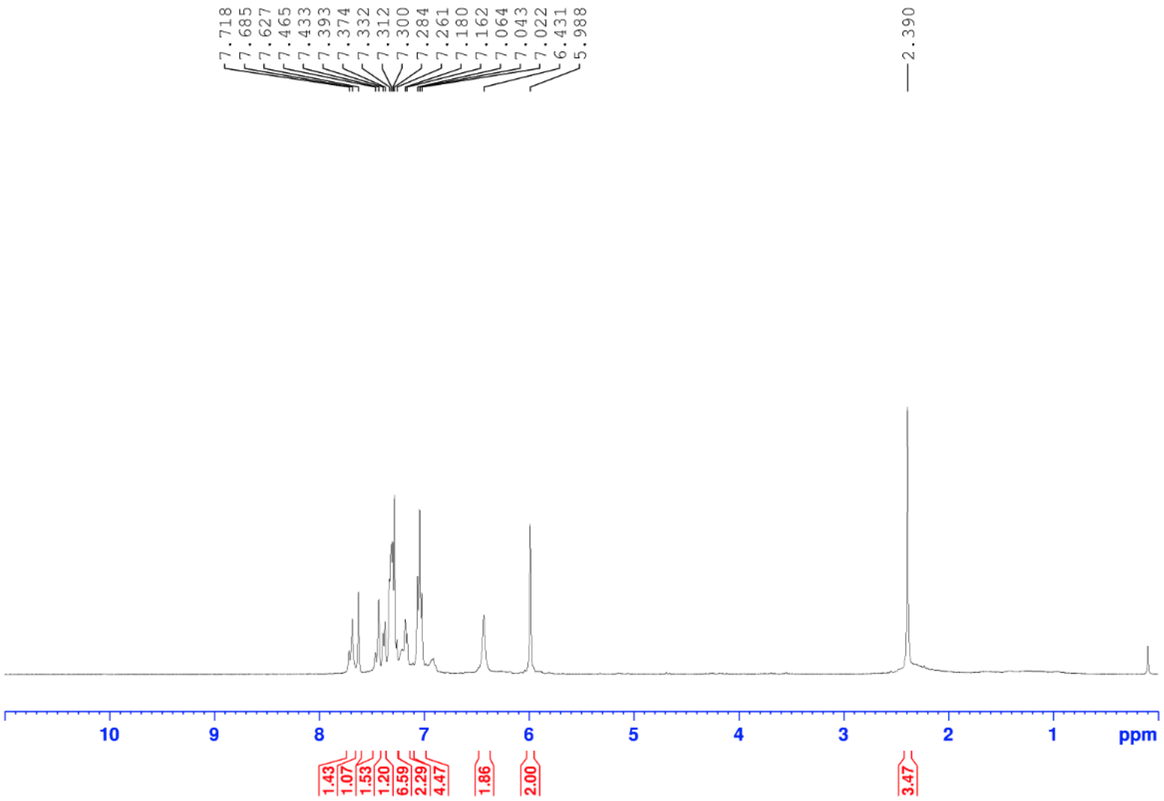

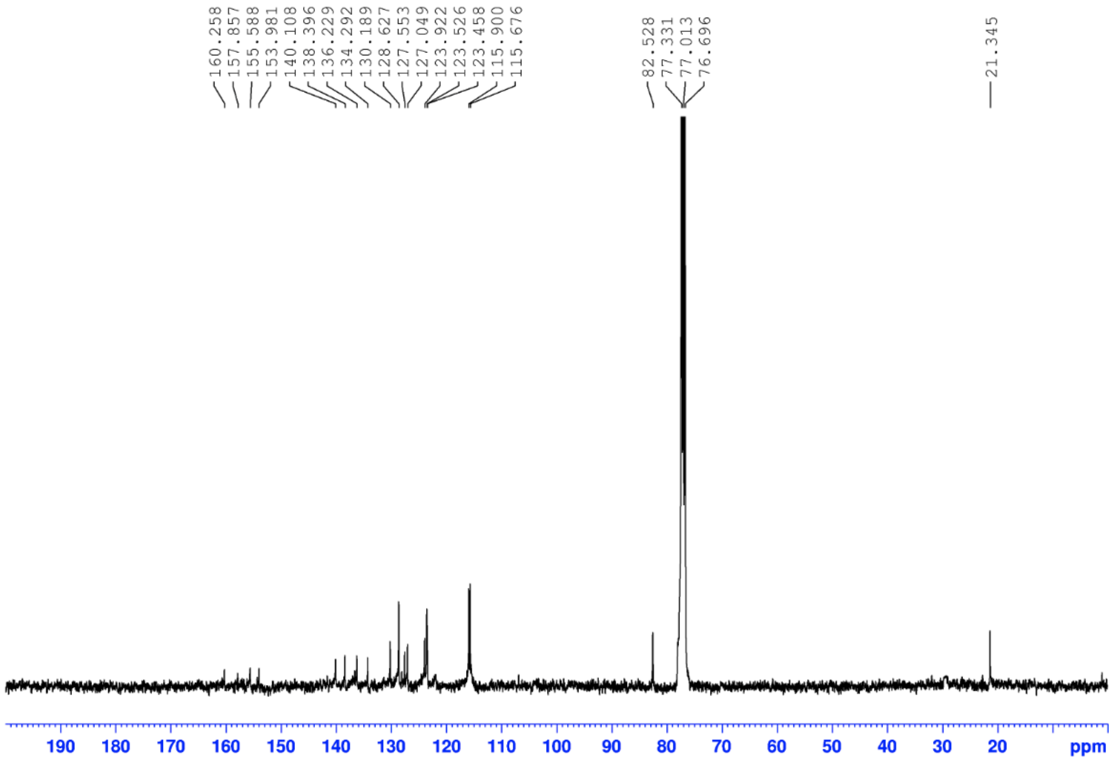

Pyridine 38a

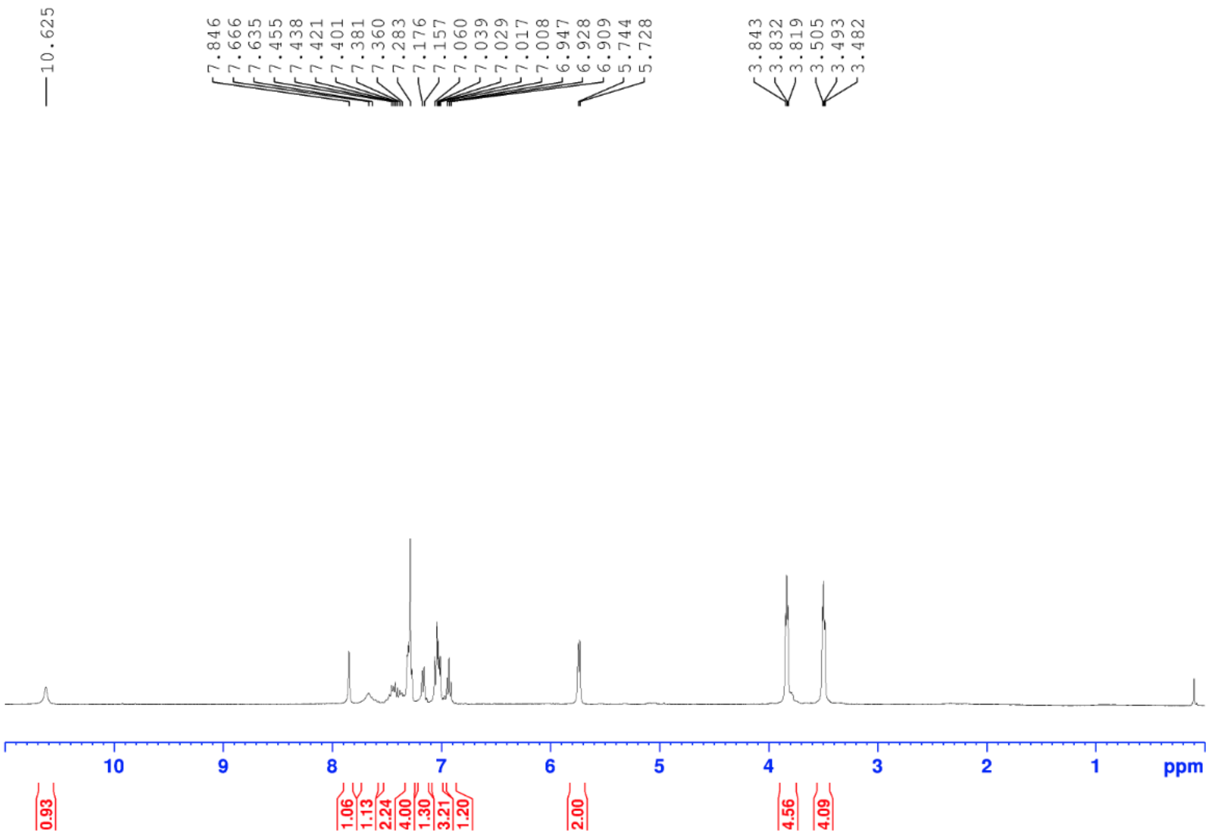

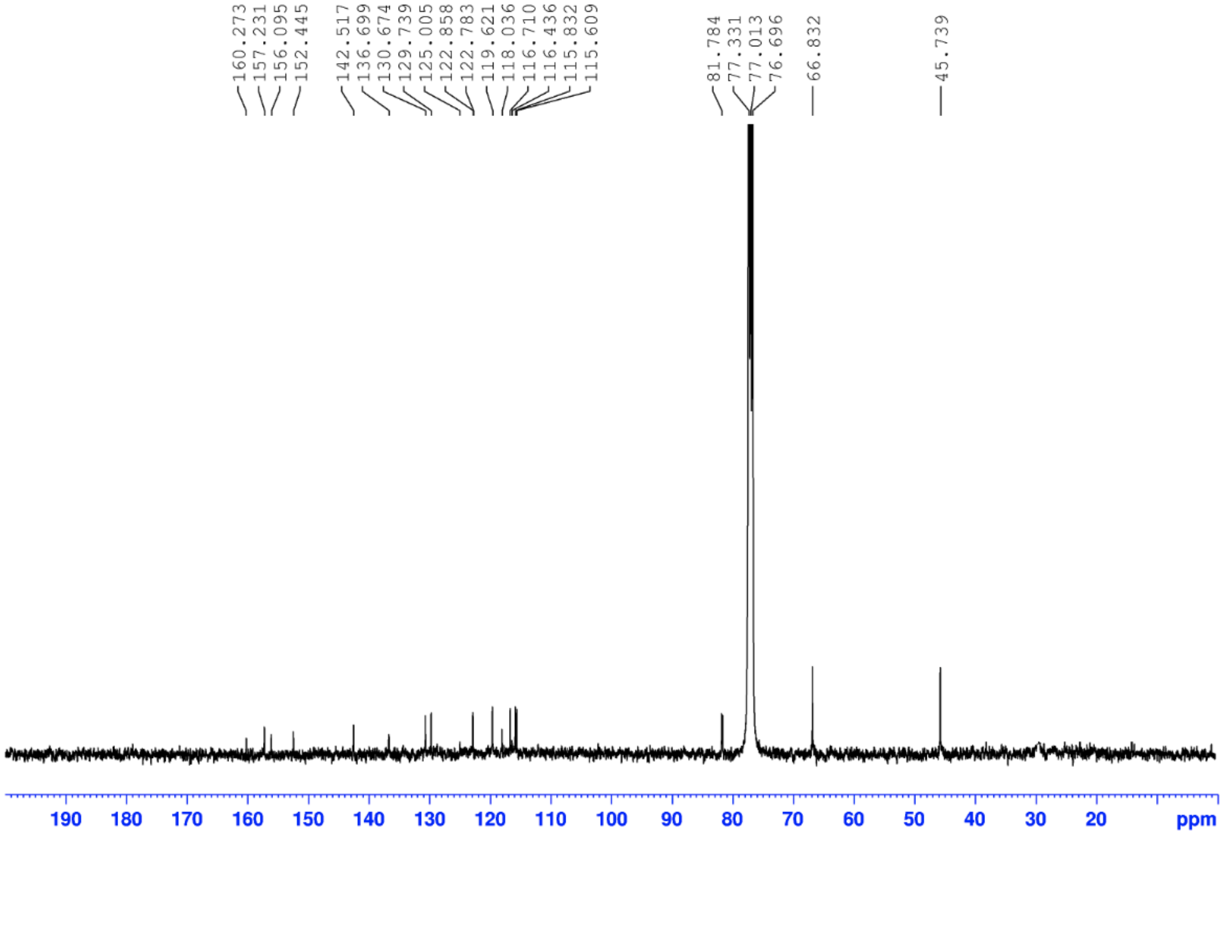

Pyridine 38b

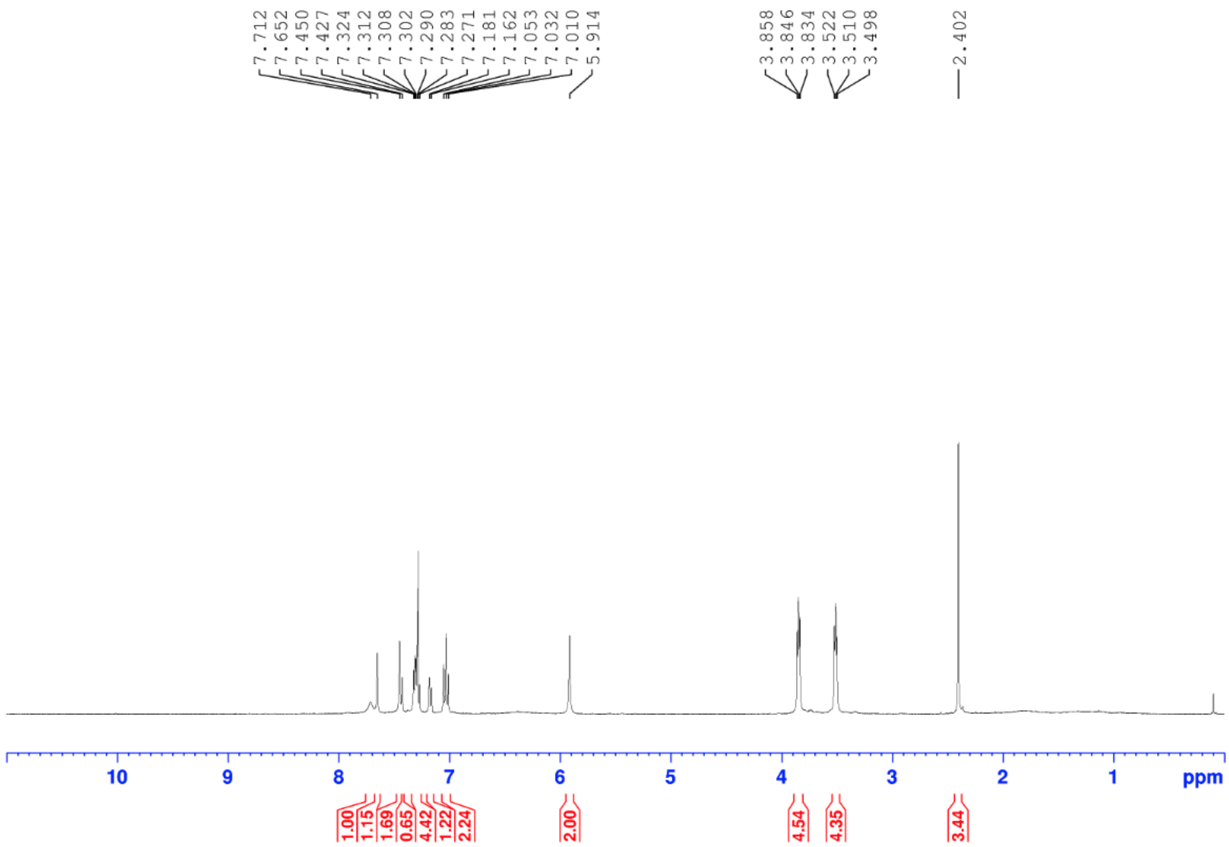

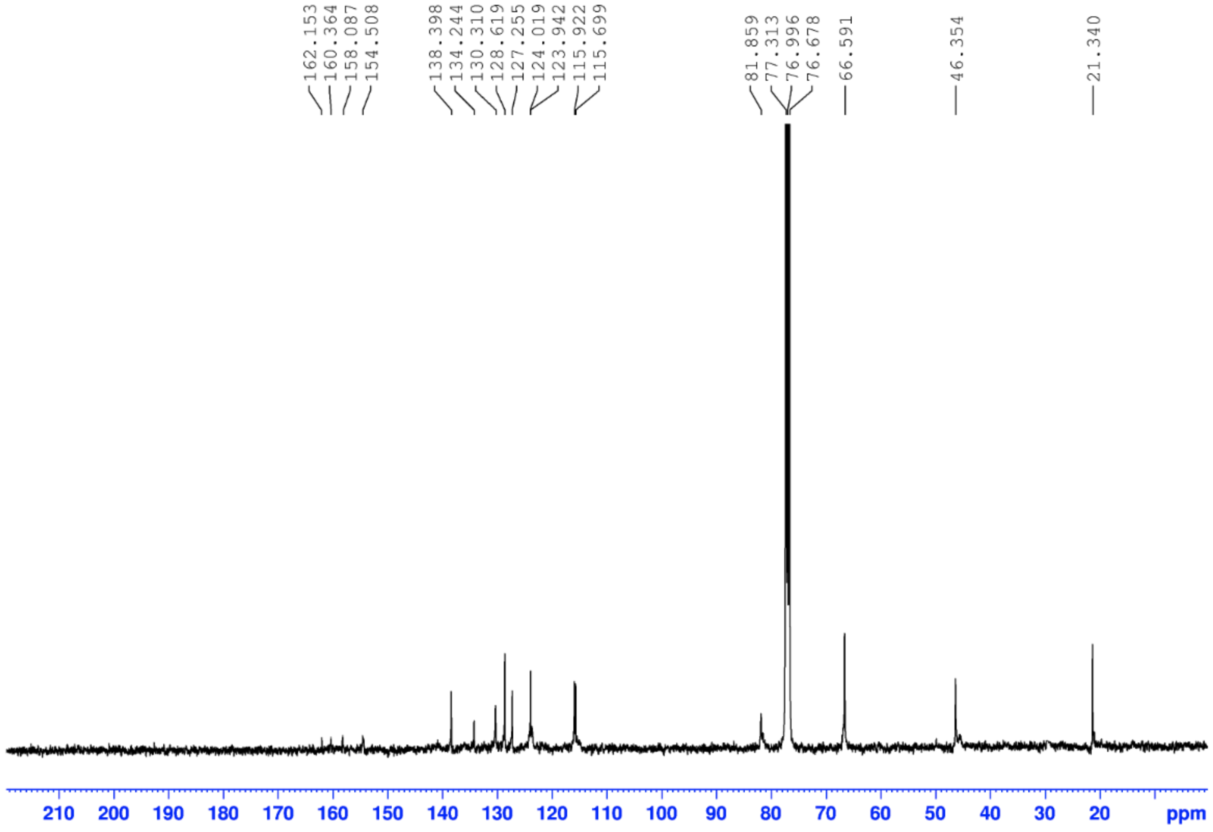

Supplement: Supplementary file 1 [file molecules-29-01452-s001.zip › molecules-2896462-supplementary.pdf]
